# The genome and sex-dependent responses to temperature in the common yellow butterfly, *Eurema hecabe*

**DOI:** 10.1186/s12915-023-01703-1

**Published:** 2023-09-25

**Authors:** Ivy H. T. Lee, Wenyan Nong, Wai Lok So, Chris K. H. Cheung, Yichun Xie, Toby Baril, Ho Yin Yip, Thomas Swale, Simon K. F. Chan, Yingying Wei, Nathan Lo, Alexander Hayward, Ting Fung Chan, Hon-ming Lam, Jerome H. L. Hui

**Affiliations:** 1 School of Life Sciences, Simon F.S. Li Marine Science Laboratory, State Key Laboratory of Agrobiotechnology, Institute of Environment, Energy and Sustainability, The Chinese University of Hong Kong, Hong Kong, China; 2 University of Exeter, Exeter, UK; 3 Dovetail Genomics, Scotts Valley, USA; 4 Agriculture, Fisheries and Conservation Department, Hong Kong, China; 5 Department of Statistics, The Chinese University of Hong Kong, Hong Kong, China; 6 School of Life and Environmental Sciences, University of Sydney, Sydney, Australia; 7 School of Life Sciences, State Key Laboratory of Agrobiotechnology, The Chinese University of Hong Kong, Hong Kong, China; 8 School of Life Sciences, State Key Laboratory of Agrobiotechnology, Institute of Environment, Energy and Sustainability, The Chinese University of Hong Kong, Hong Kong, China

**Keywords:** Butterfly, Sex-biased, Temperature, Transcriptome, Genome, Sesquiterpenoid, MicroRNA cluster

## Abstract

**Background:**

Lepidoptera (butterflies and moths) is one of the most geographically widespread insect orders in the world, and its species play important and diverse ecological and applied roles. Climate change is one of the biggest challenges to biodiversity this century, and lepidopterans are vulnerable to climate change. Temperature-dependent gene expression differences are of relevance under the ongoing climate crisis. However, little is known about how climate affects gene expression in lepidopterans and the ecological consequences of this, particularly with respect to genes with biased expression in one of the sexes. The common yellow butterfly, *Eurema hecabe* (Family Pieridae)*,* is one of the most geographically widespread lepidopterans that can be found in Asia, Africa, and Australia. Nevertheless, what temperature-dependent effects there may be and whether the effects differ between the sexes remain largely unexplored.

**Results:**

Here, we generated high-quality genomic resources for *E. hecabe* along with transcriptomes from eight developmental stages. Male and female butterflies were subjected to varying temperatures to assess sex-specific gene expression responses through mRNA and microRNA transcriptomics. We find that there are more temperature-dependent sex-biased genes in females than males, including genes that are involved in a range of biologically important functions, highlighting potential ecological impacts of increased temperatures. Further, by considering available butterfly data on sex-biased gene expression in a comparative genomic framework, we find that the pattern of sex-biased gene expression identified in *E. hecabe* is highly species-specific, rather than conserved across butterfly species, suggesting that sex-biased gene expression responses to climate change are complex in butterflies.

**Conclusions:**

Our study lays the foundation for further understanding of differential responses to environmental stress in a widespread lepidopteran model and demonstrates the potential complexity of sex-specific responses of lepidopterans to climate change.

**Supplementary Information:**

The online version contains supplementary material available at 10.1186/s12915-023-01703-1.

## Background

Lepidoptera is a large insect order that contains more than 150,000 described species of butterflies and moths and accounts for about 10% of all the described living species in the world [1]. Aside from their great biodiversity, lepidopterans also play important roles in the environment and agriculture either as pollinators, herbivores, or prey [2, 3]. Climate change, including shifting patterns of temperature and precipitation, is one of the biggest challenges to biodiversity this century. Insects such as lepidopterans may be especially vulnerable to climate change, as their body temperature can be highly correlated with the surrounding temperature [4]. Indeed, over recent years, several populations of butterflies around the globe have been affected by the changing climate and extreme temperature events [5, 6]. In general, higher temperatures result in decreases of reproductive success, changes to behaviour, and sometimes lethality in investigated species [7–11].

**Fig. 1:**
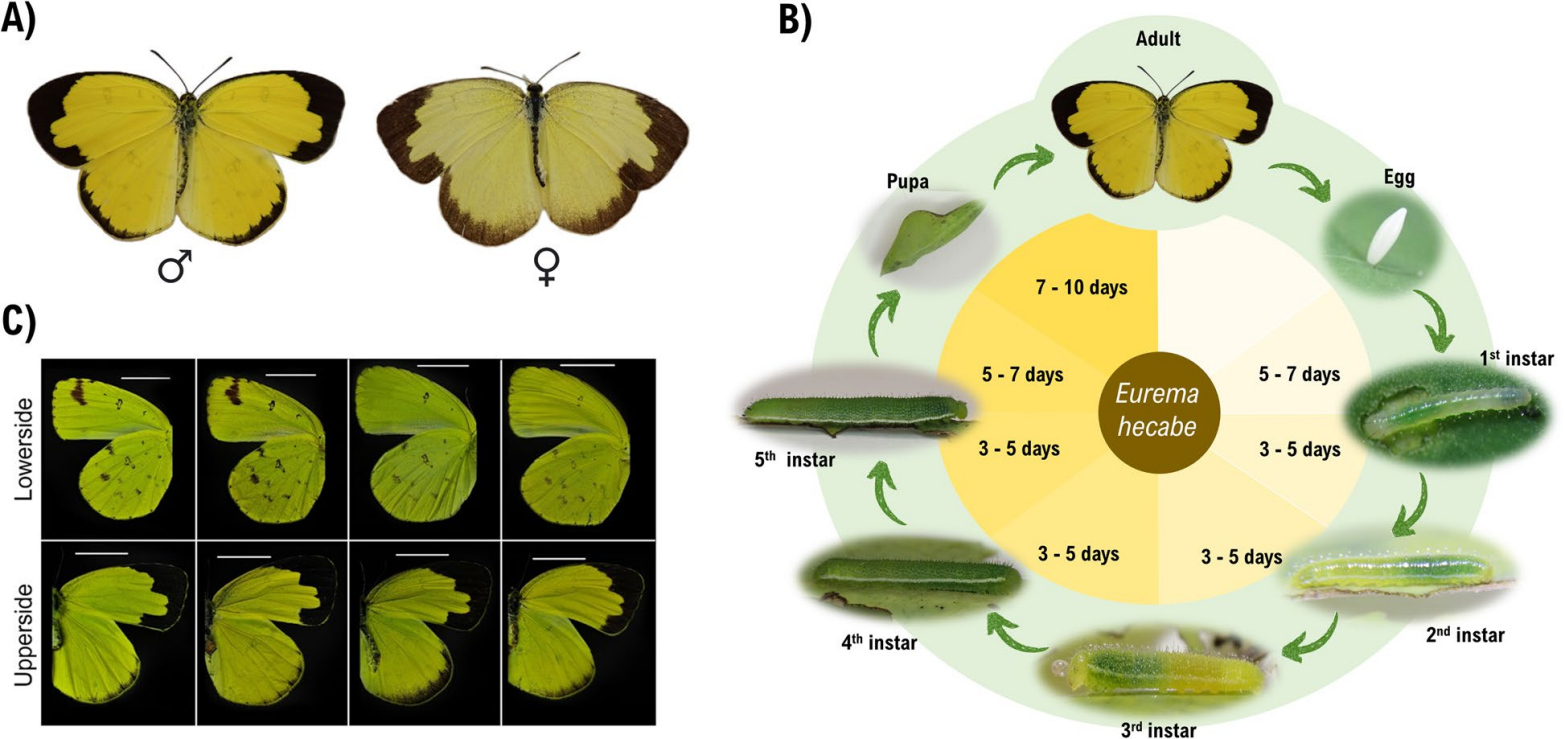
Common yellow *Eurema hecabe*. **A** Photographs of male (left) and female (right) *E. hecabe.* **B** Life cycle of *E. hecabe* cultured in the laboratory. **C** Wing morphologies of B-type *E. hecabe* collected in different seasons

Pieridae, the whites and sulphurs family of butterflies, contains about 1100 described species worldwide [12, 13]. The pierid species, the common grass yellow butterfly, *Eurema hecabe,* is an abundant and particularly widely distributed species, found in Asia, Africa, and Australia (Fig. 1A, B) [14]. Given the abundance and geographical distribution of *E. hecabe*, this species offers a useful model for understanding the effects of climate change on a common lepidopteran species across three continents.

Increasing studies have demonstrated within species divergence in traits other than morphology, such as in physiological and molecular traits. For example, environmental responses of populations from different geographical locations can differ, while the sexes of the same species can also exhibit a wide range of differences in biological processes [15]. It has been hypothesized that between sex differences in animal physiology can result in different degrees of responses to environmental change [16]. In Pieridae and Nymphalidae butterflies, protandry (males appearing earlier than females) has been reported to maximize mating opportunities of males [17–20]. Meanwhile, males and females have been shown to exhibit different immune and reproductive responses, resulting in different adaptation abilities [21–24]. However, when it comes to understanding the effects of climate change (e.g. temperature increases) on animals, most contemporary studies still treat species as having a unified response. There are also relatively few studies examining how temperature may elicit different responses between lepidopteran sexes. One study which did consider this, focusing on *Bicyclus anynana* (Nymphalidae), demonstrated significant sexual dimorphism in both eye morphology and opsin gene expressions under different temperatures [25]. Thus, while the question of sex-specific responses to climate change in lepidopterans remains largely unexplored, there is evidence that this may have relevance to important traits of evolutionary and ecological importance.

Here, we collected and sequenced the genome of *E. hecabe* and its associated *Wolbachia* strains collected in Hong Kong. In addition, a high-quality genome assembly is presented*,* together with accompanying transcriptomes from all available developmental stages (egg, first to fifth instars, pupa and adult). Adult butterflies were also cultured at different temperatures, and mRNA and microRNA transcriptomes were obtained from the two sexes, to analyse the genetic effects of variation in temperature upon *E. hecabe*.

## Results and discussion

### B-type E. hecabe in Hong Kong with high Wolbachia infection rate

**Fig. 2:**
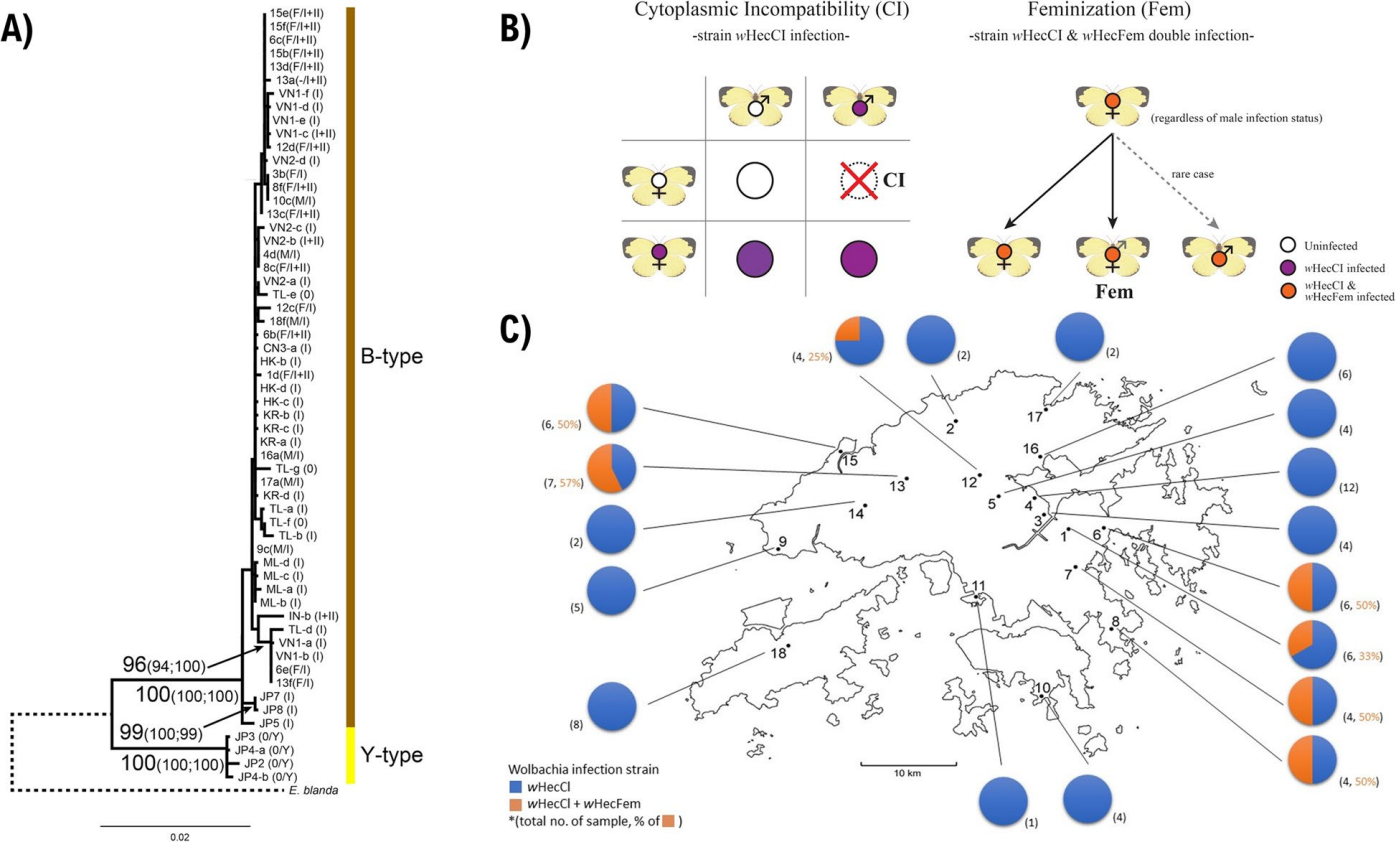
Population genetics of *E. hecabe* and their *Wolbachia* infection status in Hong Kong. **A** Phylogenetic analysis of concatenated COI, COIII, and ND5 gene in this study with sequences from other countries (sequences from Narita et al., 2007a), showing all samples collected in this study were found to be B-type. **B** Schematic diagram showing the reproductive phenotypes of *E. hecabe* infected with different *Wolbachia* strains. For details, please refer to the main text. **C** The proportion of wHecCI infection and wHecCI + wHecFem double infection are shown in pie charts

Previous studies have identified two sibling species, the B- and Y-types, in *E. hecabe* based on the mitochondrial markers: cytochrome *c* oxidase subunit I (COI), cytochrome *c* oxidase subunit III, and NADH dehydrogenase subunit 5 (e.g. [26]). The B-type is generally widespread in tropical and subtropical areas, while the Y-type is usually restricted to temperate areas in the northern Japan, with the exception of a Y-type individual collected in Hong Kong in 1995 [26]. Variations among different aspects of the biology of these sister species have been observed. For example, morphological variation including polymorphic forms of wing patterns have been found during different seasons and in different geographical locations in Japan [27, 28], and the colour of the forewing fringe on the B-type is brown while it is yellow on the Y-type [29]. Furthermore, behavioural and ecological variation, such as host plant utilization [30], larval feeding response [30], and mate choice [31] has also been reported between the two genetic subtypes. Due to the above distinctions, Y-type *E. hecabe* is now reclassified as the separate species *E. mandarina*, while the B-type *E. hecabe* is regarded as a monophyletic and homogenous lineage [26]. Among the 89 COI sequences (1326 bp) we collected, 40 polymorphic sites were identified with 24 being parsimony-informative. The wing morphology of individuals collected from different seasons were also compared; however, no identifiable differences were observed (Fig. 1C). Using COI sequences together with sequences deposited in GenBank, all collected butterflies were identified as B-type *E. hecabe,* supported by high bootstrap values or probabilities in all phylogenetic analyses (94% in maximum-likelihood, 95% in neighbour-joining, and 100% in Bayesian inference, Fig. 2A).

The endosymbiont *Wolbachia* represents one of the most abundant bacteria present in butterflies [32]. There are at least four major subgroups/strains of *Wolbachia* (A–D), with strains A and B thought to have diverged ~ 60 million years ago and be confined to arthropods [33]. Two *Wolbachia* B subgroup strains, *w*HecCI (strain I) and *w*HecFem (strain II), have been found to infect *Eurema* butterflies*,* with profound influences on host sex determination well documented in *Eurema mandarina* [32, 34, 35]. Infection with *w*HecCI occurs both in isolation and together with *w*HecFem, while infection with *w*HecFem appears to only occur together with *w*HecCI [36]. Infection with *w*HecCI induces cytoplasmic incompatibility (CI) when an infected male mates with an uninfected female [36, 37] (Fig. 2B). Meanwhile, in *w*HecFem infected lineages, the W chromosome has been lost [34, 35], and associated loss of the female-determining W chromosome is functionally compensated by *w*HecFem [34, 38]. Additionally, a meiotic drive mechanism appears to prevent inheritance of the maternal Z chromosome due to non-random segregation of the sex chromosomes [34, 35]. Thus, in *w*HecFem infected individuals, the production of ZZ progeny is inhibited resulting in only 0Z eggs being produced (with a paternally inherited Z), and the resultant offspring are feminized by *w*HecFem to become functional females, effectively leading to all female progeny [34, 35]. Among 89 analysed samples, all were found to be infected by *Wolbachia* strain *w*HecCI, while 18 (20.2%) were co-infected with *Wolbachia* strain *w*HecFem (Fig. 2C).

### High-quality E. hecabe genome, macrosynteny, and gene expansion

**Fig. 3:**
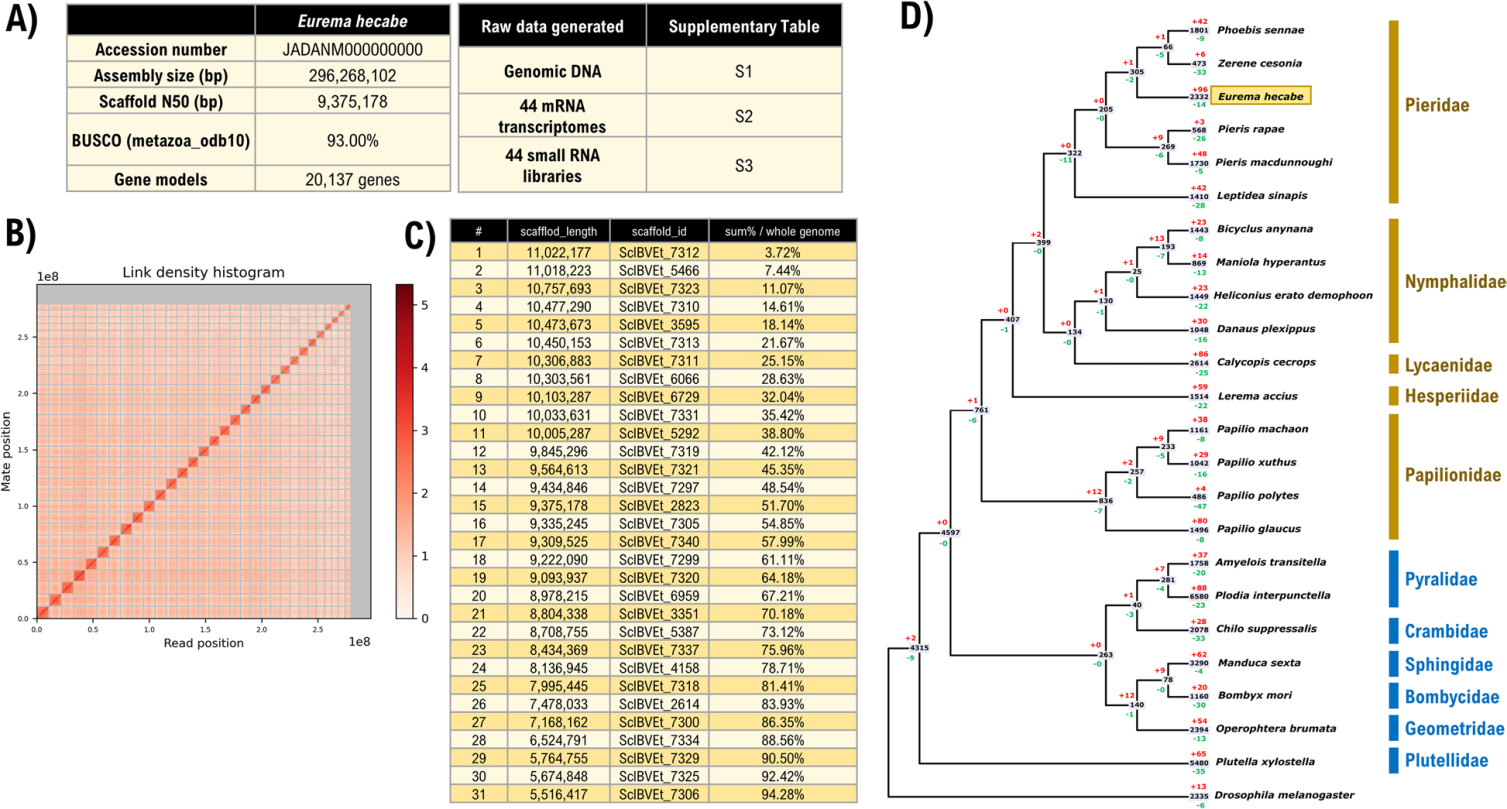
Genomic analyses of *E. hecabe* genome. **A** Tables of genome statistics and resources generated in this study. **B** Link density histogram of the *E. hecabe* genome. **C** Information of the 31 pseudochromosomes. **D** Gene family expansion and contraction in insects. Red and green colours indicate the number of significantly (*p* < 0.05) expanded ( +) or contracted (-) gene families at each node, respectively

We generated a high-quality chromosome-level genome assembly of *E. hecabe* (2*n* = 62), for which 94.28% of the genomic sequences are contained on 31 pseudomolecules (~ 296.3 Mb) (Fig. 3A, B), which is compatible with the estimated genome size of 314.7 Mb by k-mer 21 (Additional file 1: Figure S1 & 2). The BUSCO (Benchmarking Universal Single-Copy Orthologs) score for complete genes is 93%, and the scaffold N50 and L50 of the genome are 15 Mb and 9.375 Mb, respectively (Fig. 1C).

The *E. hecabe* genome contains ~ 20% repetitive sequences, with 999 distinct transposable element (TE) classifications identified by our annotation (Additional file 1: Figure S3, Additional file 2). The dominant TE type is rolling circle elements (8.7%), with the next most abundant TE type being unclassified elements (4.6%), followed by LINEs (4.5%). There are few LTR elements (0.8%) and very few SINEs (0.001%) present in the genome (Additional file 2). LINEs are the most diverse TE type in the *E. hecabe* genome, with 385 distinct LINE families identified by our analysis. A repeat landscape analysis suggests a gradual increase in TE activity over time, followed by a slight recent decline in activity (Additional file 1: Figure S3). Consistent with their abundance in the *E. hecabe* genome, rolling circle elements and LINEs are the most recently active TEs, but there appears to have been a considerable very recent increase in DNA and LTR element activity (Additional file 1: Figure S3).

Using the transcriptomic data, we predicted a total of 20,137 gene models, including 18,197 protein coding genes and 1940 tRNA genes in this genome, which is in the range similar to other published Pieridae genomes (Fig. 3C, D). The pseudochromosomes of *E. hecabe* displayed significant one-to-one relationships against the genomic scaffolds in other butterflies (Additional file 1: Figure S2). Lineage blocks were also visible when compared with the fruit fly *D. melanogaster*, suggesting that the genome architecture of these lineages had not undergone active chromosomal rearrangements from their last common ancestor.

### Conserved and novel microRNAs in E. hecabe

**Fig. 4:**
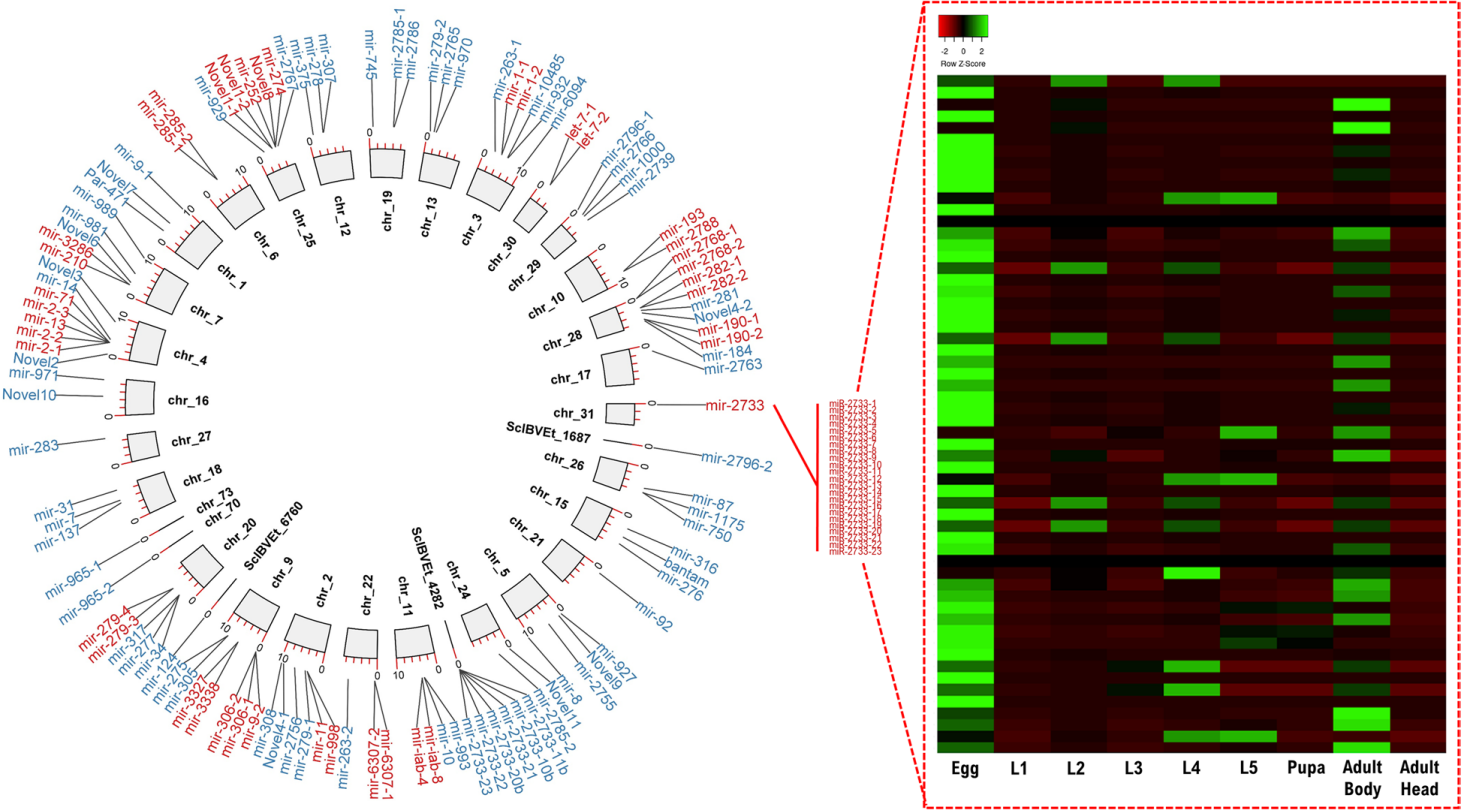
Conserved and novel microRNA loci annotated in the *E. hecabe* genome. Blue colour indicated non-clustered microRNAs while red colour indicated the clustered microRNAs

MicroRNAs are 21–23 nucleotides of noncoding RNAs that regulate gene expression post-transcriptionally and have been suggested to be a potential contributor to the evolution of arthropod developmental processes and morphologies (e.g. [39–42]). Previous sequencing of small RNA from lepidopterans identified a burst of microRNA innovation in these lineages [43]. Here, we sequenced the small RNA transcriptomes from 8 developmental stages, including egg, first to fifth instars, pupa, and adult of *E. hecabe* (Fig. 1D). Among all microRNA precursor candidates identified in *E. hecabe*, a total of 130 microRNAs were identified (Fig. 4). Similar to a previous study [43], we also identify novel or lineage-specific microRNAs, finding 12 such sequences in *E. hecabe*. Further, a total of 118 conserved microRNAs, including 43 lepidopteran lineage-specific microRNAs, which were previously thought to be species-specific, were identified (Fig. 4). An interesting phenomenon is that a cluster of tandemly duplicated miR-2733 on chromosome 31 was significantly expanded in this genome similar to other lepidopterans such as the moths *B. mori* and *Heortia vitessoides* [44, 45], and members of this miR-2733 cluster are relatively highly expressed in the egg and adult bodies, but not in the pupae stages (Fig. 4).

### Sex-specific responses upon temperature change

Sexual dimorphism refers to the phenomenon of females and males of the same species exhibiting a wide range of differences in various biological processes. The availability and feasibility of transcriptomic profiling made new genome-wide discovery of underlying mechanisms possible. One such discovery is sex-biased gene expression, where morphological differences between sexes of the same species are caused by differential expression of genes that are present in both sexes [46]. In lepidopterans, *BmOr19* has been shown to have a female-biased expression in the antennae of *Bombyx* for seeking host plants, while female-biased expression of *ultraviolet opsin gene 1* (*UVRh1*) in *Heliconius* might also link to searching of oviposition sites [47–49]. Deep sequencing of microRNAs in different animals is now also showing microRNAs exhibiting sex-biased expression in different groups of animals. In insects, sex-biased microRNA expression has been revealed in different developmental stages, for instance, in the flies *Bactrocera dorsalis* and *D. melanogaster* [50–54], the beetle *Tribolium castaneum* [55], the termite *Reticulitermes speratus* [56], the plant hopper *Sogatella furcifera* [57], the wasp *Ceratosolen solmsi* [58], the bee *Apis mellifera* [59], and the mosquito *Anopheles gambiae* [60]. Studies have shown that environmental cues can also relate to sex-biased protein coding gene expression. For example, 11 microRNAs showed sex-biased expression patterns in the red flour beetle *Tribolium castaneum* during stresses including temperature, starvation, and bacterial infection [55]. Nevertheless, sex-biased genes and microRNAs responding to environmental cues such as temperature changes in lepidopterans remain poorly known.

**Fig. 5:**
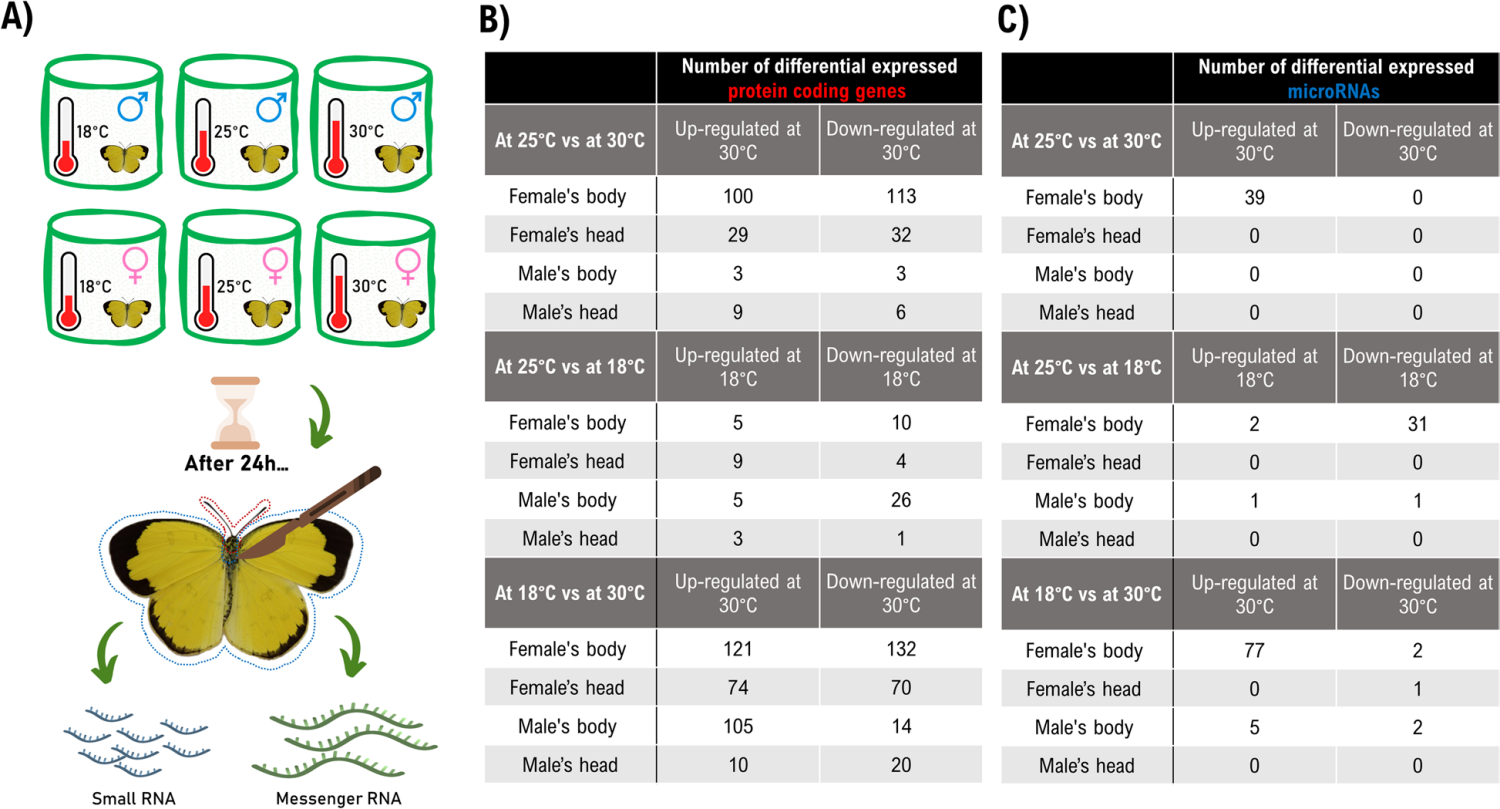
Transcriptomes of heads and bodies of male and female adults of *E. hecabe* treated at different temperatures. **A** Schematic diagram showing the experimental design. **B** Table summarizing the number of differentially expressed protein-coding genes in different tissues of different sexes at different temperatures. **C** A table summarizing the number of differentially expressed microRNAs in different tissues of different sexes at different temperatures

**Fig. 6:**
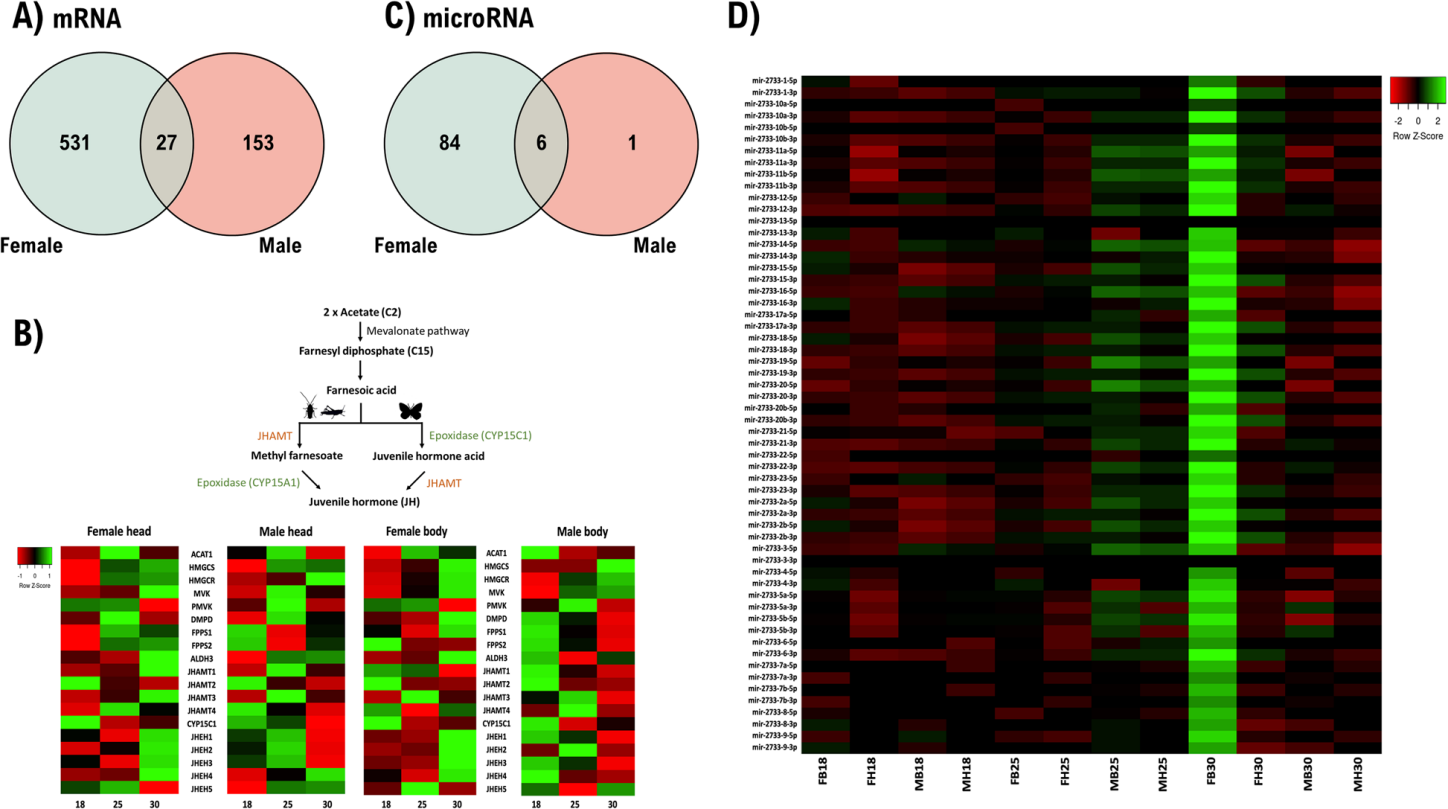
Sex-specific responses of different tissues under different temperatures of *E. hecabe*. **A** A Venn diagram showing the total number of differentially expressed protein-coding genes identified in both sexes under different temperatures. **B** Schematic diagram showing the canonical biosynthetic pathway of sesquiterpenoid hormones in insects (upper), and the heatmaps showing expression of sesquiterpenoid biosynthetic pathway genes in different tissues at different temperatures. **C** A Venn diagram showing the total number of differentially expressed microRNAs identified in both sexes under different temperatures. **D** Heatmap showing expression of members in the miR-2733 cluster (F, female; M, male; B, body; H, head; 18, at 18 °C; 25, at 25 °C; 30, at 30 °C)

Here, we treated male and female adult *E. hecabe* at three different temperatures and revealed mRNA and sRNA transcriptomic responses in their heads and bodies (Fig. 5A; Additional file 1: Figure S4). The temperature ranges were chosen based on those that this species will experience in its natural environment. As shown in Fig. 5B, the protein coding gene responses in heads and bodies between males and females are largely similar, except between 25 °C and 30 °C, where there are much more differentially expressed genes in different tissues of females than males. Comparison of all the differentially expressed genes between males and females found that there is only a subset of genes in common (Fig. 6A; Additional file 1: Figure S5 & S6; Additional file 3; Additional file 4), suggesting that the genetic responses of *E. hecabe* to temperature changes are largely sex-specific. Gene pathway enrichment analyses suggested that different gene ontology and pathways are enriched in different contexts between sexes (Additional file 5; Additional file 1: Figure S7 – S9).

To understand the above observation, we further investigated the expression of sesquiterpenoid pathway genes, as sesquiterpenoids such as juvenile hormone regulate development, physiology, metamorphosis, and reproduction in insects [61–63]. In the *E. hecabe* genome, we were able to identify all genes in the mevalonate and juvenile hormone biosynthesis pathways (Fig. 6B; Additional file 6). It is worth noting that there were two copies of farnesyl diphosphate synthase (FPPS) identified in *E. hecabe*. Multiple copies of FPPS are commonly reported in other butterflies and moths, including *Bombyx mori*, *Choristoneura fumiferana*, *Danaus plexippus*, *Helicoverpa armigera*, *Mythimna unipuncta*, *Papilio glaucus*, *Papilio xuthus*, and *Pieris rapae* [64–68]. In addition, no CYP15A1 could be identified in *E. hecabe,* similar to other lepidopterans [63]. In the heads of females and males *E. hecabe* treated at different temperatures, we found that most sesquiterpenoid biosynthetic pathway genes have similar differential responses, with the exception of a few genes, such as mevalonate kinase (MVK), which was upregulated in the female head at 25 °C but downregulated in male head at 30 °C (Fig. 6B). This result suggests the hormonal system responds differently in the different sexes of lepidopterans upon temperature changes.

Considering microRNAs, similar to the responses of protein coding genes, we observed sex-biased responses in both the heads and bodies of the females and males of *E. hecabe*, and there are many more differentially expressed microRNAs in different tissues of females than males, similar to the picture for protein genes (Figs. 5C and 6C; Additional file 1: Figure S10; Additional file 7). As mentioned above, a lepidopteran miR-2733 cluster is revealed in the *E. hecabe* genome, and members of insect microRNA cluster have been hypothesized to reinforce and modify the selection force of cluster regulation and gene regulatory network of existing microRNAs [42]. We found that members of miR-2733 were upregulated in temperature 30 °C only in the body of female *E. hecabe*, but not in other treatments (Fig. 6D). Although the way in which this microRNA cluster contributes to the adaptation of *E. hecabe* to its environment remains to be functionally tested, we have demonstrated the microRNAs and cluster of microRNAs respond to environmental cues differently between sexes, rather than as a largely unified or similar response.

In the analysis of neuropeptides expression profiles between the head and body transcriptomes, we found that there is a clear discrepancy (Additional file 8). Meanwhile, most of the neuropeptides were expressed in the early developmental stages (i.e. egg and 1st instar), and some were specifically expressed higher in the adult stage, including adipokinetic hormone (AKH), crustacean hyperglycemic hormone (CHH), diapause hormone/PBAN/pyrokinin, insulin-like peptide, ion-transport peptide, neuroparsin, partner of bursicon (pburs), prothoracicotropic hormone (PTTH), short neuropeptide F (sNPF), SIFamide, and Trissin 2 (Additional file 1: Figure S11). These neuropeptides have been found to be important regulators in lepidopterans, for instance, insulin controls the seasonal plasticity, development, and diapause in some species [69–71], while other hormones such as ecydosone have been shown to drive seasonality plasticity in some species of butterflies [72, 73]. Furthermore, we discovered several sex-biased neuropeptides, including CHH, IMFamide, ion-transport peptides, and pburs (red boxes highlighted in Additional file 1: Figure S11). How these different neuropeptides function in butterflies remains to be explored.

**Fig. 7:**
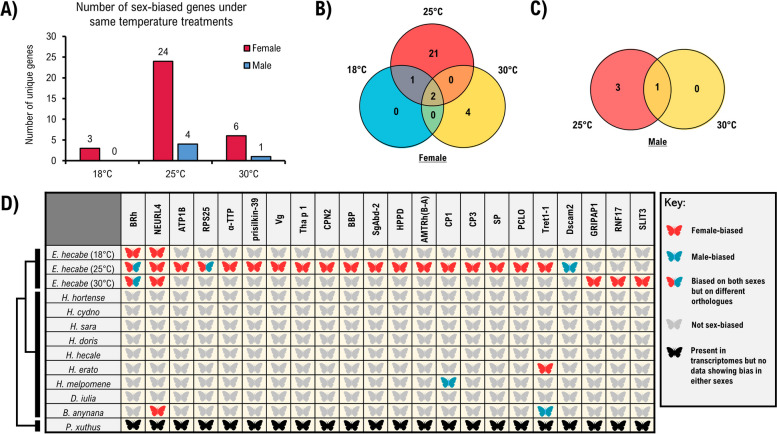
Sex-specific responses of *E. hecabe* head tissue compared to other different published butterfly transcriptomics. **A** Bar plot showing the number of sex-biased genes under same temperature treatments in both sexes of *E. hecabe*. **B** Venn diagram illustrating the female-biased genes under different temperature (18 °C, 25 °C, and 30 °C) treatments. **C** Venn diagram illustrating the male-biased genes under different temperature (25 °C and 30 °C) treatments. **D** Comparative analysis showing the sex-biased genes in *E. hecabe* and other related published sex-biased transcriptomic studies. The expression data for *Bicyclus anynana* is extracted from Ernst and Westerman (2021). BRh, blue (B-) sensitive rhodopsin; NEURL 4, neuralized-like protein 4; ATP1B, sodium/potassium-transporting ATPase subunit beta-2; RPS25, 40S ribosomal protein S25; α-TTP, alpha-tocopherol transfer protein; Vg, vitellogenin-like; Tha p 1, allergen Tha p 1-like; CPN2, carboxypeptidase N subunit 2-like; BBP, bilin-binding protein-like; SgAbd-2, endocuticle structural glycoprotein SgAbd-2-like; HPPD, 4-hydroxyphenylpyruvate dioxygenase-like; AMTRh(B-A), ammonium transporter Rh type B-A; CP1, cuticle protein 1; CP3, cuticle protein 3-like; SP, serine protease snake-like; PCLO, protein piccolo; Tret1-1, facilitated trehalose transporter Tret1-like; Dscam2, Down syndrome cell adhesion molecule-like protein; GRIPAP1, GRIP1-associated protein; RNF17, RING finger protein 17; SLIT3, slit homolog 3 protein-like

Given there is data available on sex-biased gene expression for a range of butterfly models [49, 74–77], we then compared sexual dimorphic gene expression across lineages (Additional file 9). In our analysis of *E. hecabe* head transcriptomes, more differential expression of sex-biased genes was found when butterflies were reared at 25 °C compared to 18 °C and 35 °C, with the number of sex-biased genes being 24 and 4 genes in females and males, respectively (Fig. 7A). Only 2 genes (*blue-sensitive rhodopsin*, *BRh* and *neuralized-like protein 4*, *NEURL4*) were found to be consistently expressed in a sex-biased manner in females at different temperatures (Fig. 7B), while only 1 gene was found in the males (another form of *BRh*) (Fig. 7C). Surprisingly, we identified a list of genes with female-biased expression in *H. hecabe,* but not in other available butterflies, while *NEURL4* also displayed a similar pattern in *Bicyclus anynana* (Fig. 7D). In addition, we observed a pattern of opposite sex-specific expression of *facilitated trehalose transporter Tret1-like*, *Tret-1* among one set of species, while this gene is female-biased in *E. hecabe* and *Heliconius erato*, it is male-biased in *B. anynana*. While efforts have been made here to understand sex-biased gene expression in butterflies, our current understanding of this topic is still in its infancy.

This study established a high-quality genome assembly and transcriptomic resources for a butterfly species *E. hecabe* that can be commonly found in three continents. Differential genetic responses between different sexes upon environmental parameter changes were tested and revealed. It is envisioned that this established model can be easily adopted and used to understand other aspects of lepidopteran biology and responses to climate change.

## Conclusions

We provide high-quality genomic resources for one of the most common widely distributed butterfly species, *Eurema hecabe*, the common grass yellow, which can be found across three continents (Asia, Africa, Australia). For the first time, we provide a chromosomal-level genome assembly for this species, as well as 87 new mRNA and small RNA transcriptomes. In addition, we demonstrate considerable feasibility to work on this species as a model for a range of pertinent questions in evolution and ecology. We revealed that different tissues of different sexes of this butterfly species respond differently across temperatures, including differences in the expression of key hormonal genes as well as noncoding microRNAs. The foundation set up in this study will be useful for future molecular ecology studies on the following aspects, including but not limited to population genomics to reveal their migratory patterns, subpopulation and speciation event, climate change effects on them, and compare to other well established butterfly species found on the same and other continents.

## Methods

### Field collections and detection of Wolbachia strain wHecCI and wHecFem

Eighty-nine individuals including 79 adults and 10 larvae of *E. hecabe* were collected from different locations in Hong Kong from 2014 to 2015 (Additional file 10). Sampling was undertaken at geographical sites where *E. hecabe* is common. An adult of *E. blanda* was collected at Wong Nai Tun (22.415, 114.022) and used as the outgroup in phylogenetic analyses. The sex of the adult individuals was determined by the sexually dimorphic wing patterns and genital morphology at the end of abdomen, while the presence/absence of testes was used to determine sex in the last instar larvae. All specimens were labelled with collection locality, GPS coordinates, date, and stored at − 20 °C in the laboratory before further processing. Details of the collection sites are listed in Additional file 10. Genomic DNA was extracted from the abdominal/thoracic muscle of adults or gut-removed abdominal tissue from larvae using PureLink Genomic DNA Mini Kit (Invitrogen). The mitochondrial COI gene, encoding cytochrome *c* oxidase subunit I, was amplified using primers 5’-ATTCAACCAATCATAAAGATAT-3’ and 5’-ATCAGAATAACGTCGAGGTAT-3’ following a previous study [78]. The infection status of *Wolbachia* was first screened by performing PCR with 16S rRNA marker using primers (5’-TTGTAGCCTGCTATGGTATAACT-3’) and (5’-GAATAGGTATGATTTTCATGT-3’) [79], and specific strains were further identified using primers wsp81F, 5’-TGGTCCAATAAGTGATGAAGAAAC-3’ and WHecFem1, 5’-ACTAACGTCGTTTTTGTTTAG-3’ for strain *w*HecCI; and primers WHecFem2, 5’-TTACTCACAATTGGCTAAAGAT-3’ and wsp691R, 5’- AAAAATTAAACGCTACTCCA-3’ for strain *w*HecFem [80]. PCR was performed as follows: 95 °C for 3 min, followed by 35 cycles of 95 °C for 1 min, 58 °C for 1.5 min, and 72 °C for 1.5 min, and a final extension at 72 °C for 7 min. PCR products were subjected to gel electrophoresis.

### Butterfly culture

Adult *E. hecabe* were collected from different locations in Hong Kong during 2019 to 2021, including The Chinese University of Hong Kong at Ma Liu Shui (22.421, 114.208), Tai Po Kau (22.422, 114.178), Shing Mun Country Park (22.385, 114.143), Sai Kung (22.419, 114.349), Hok Tau (22.489, 114.182), and Luk Keng (22.520, 114.214). Animals were kept in a net cage (width: 60 cm, length: 50 cm, height: 90 cm) at room temperature (~ 23 °C), with a humidity 40% and 14:10 h of light–dark cycle; 10% honey solution was prepared from commercially available “Bee easy wild flower honey” (Langnese, Germany) and provided to the animals. Fresh leaves of *Senna surattensis* were collected from The Chinese University of Hong Kong and were provided to the adult animals in cage for oviposition. Photo documentation of animals was carried out with a digital camera (Olympus Tough TG-6).

### Genome sequencing

Sample for genome sequencing originates from a single *E. hecabe* individual within the established laboratory culture (Fig. 1A, B), with genomic DNA (gDNA) extracted using the PureLink Genomic DNA Mini Kit (Invitrogen) following the manufacturer’s protocol. Extracted gDNA was subjected to quality control using a Nanodrop spectrophotometer (Thermo Scientific) and gel electrophoresis. Qualifying samples were sent to Novogene and Dovetail Genomics for library preparation and sequencing. The resulting library was sequenced on Illumina HiSeq X platform to produce 2 × 150 paired-end sequences. The length-weighted mean molecule length is 10,226.69 bases, and the raw data can be found at NCBI’s Small Read Archive (SRR12799420) (Additional file 10).

### Chicago library preparation and sequencing

A Chicago library was prepared from another *E. hecabe* individual, which was collected from the established laboratory culture as described previously [81]. Briefly, ~ 500 ng of HMW gDNA (mean fragment length = 85 kbp) was reconstituted into chromatin in vitro and fixed with formaldehyde. Fixed chromatin was digested with DpnII, the 5’ overhangs filled in with biotinylated nucleotides, and free blunt ends were ligated. After ligation, crosslinks were reversed, and the DNA purified from protein. Purified DNA was treated to remove biotin that was not internal to ligated fragments. The DNA was then sheared to ~ 350 bp mean fragment size and sequencing libraries were generated using NEBNext Ultra enzymes and Illumina-compatible adapters. Biotin-containing fragments were isolated using streptavidin beads before PCR enrichment of each library. The libraries were sequenced on an Illumina HiSeq X to produce 46,027,927 2 × 150 bp paired end reads (1–100 kb pairs) (Additional file 10).

### Dovetail HiC library preparation and sequencing

An additional Dovetail HiC library was prepared with the same individual used in Chicago library preparation. Briefly, for each library, chromatin was fixed in place with formaldehyde in the nucleus and then extracted fixed chromatin was digested with the restriction enzyme DpnII, the 5’ overhangs were filled in with biotinylated nucleotides, and free blunt ends were ligated. After ligation, crosslinks were reversed, and the DNA purified from protein. Purified DNA was treated to remove biotin that was not internal to ligated fragments. The DNA was sheared to ~ 350 bp mean fragment size and sequencing libraries were generated using NEBNext sUltra enzymes and Illumina-compatible adapters. Biotin-containing fragments were isolated using streptavidin beads before PCR enrichment of each library. The libraries were sequenced on an Illumina HiSeq X sequencer to produce 46,890,786 2 × 150 bp paired end reads (10–10,000 kb pairs) (Additional file 10).

### Transcriptome sequencing

Total RNA from different developmental stages and adult body tissues (head and body) were isolated using a combination method of cetyltrimethylammonium bromide (CTAB) pre-treatment [82] and mirVana™ miRNA Isolation Kit (Ambion) following the manufacturer’s instruction (details can be found in Additional file 10). Different developmental stages RNA (one individual for each stage) was obtained for sequencing in order to better annotate the gene models of this newly established genome. Total RNA from different tissues were extracted from different sexes of butterflies at different temperature, in order to test whether different tissues of males and females respond similarly/differently at different temperatures. The extracted total RNA was subjected to quality control using a Nanodrop spectrophotometer (Thermo Scientific), gel electrophoresis, and an Agilent 2100 Bioanalyzer (Agilent RNA 6000 Nano Kit). Qualifying samples underwent library construction and sequencing at Novogene; polyA-selected RNA-Sequencing libraries were prepared using TruSeq RNA Sample Prep Kit v2. Insert sizes and library concentrations of final libraries were determined using an Agilent 2100 bioanalyzer instrument (Agilent DNA 1000 Reagents) and real-time quantitative PCR (TaqMan Probe), respectively. Details of the sequencing data can be found in Additional file 10.

### Genome assembly

Chromium WGS reads were used to make a de novo assembly using Supernova (v 2.1.1) with default parameters (raw coverage = 54.18 ×) (Additional file 1: Figure S11). The Supernova output pseudohaplotype assembly, shotgun reads, Chicago library reads, and Dovetail HiC library reads were used as input data for HiRise, a software pipeline designed specifically for scaffolding genome assemblies using proximity ligation data [83]. An iterative analysis was conducted as follows. First, Shotgun and Chicago library sequences were aligned to the draft input assembly using a modified SNAP read mapper (http://snap.cs.berkeley.edu). The separations of Chicago read pairs mapped within draft scaffolds were analysed by HiRise to produce a likelihood model for genomic distance between read pairs, and the model was used to identify and break putative misjoins, to score prospective joins, and make joins. In order to identify a misjoin, the likelihood model was used to compute the log likelihood change gained by joining both sides of each position of each contig in the initial assembly. A score would then be calculated. If this score fell below the threshold values over a maximal internal segment of an input contig, the segment was thus defined as a “low support” segment and a break was introduced [83]. After aligning and scaffolding Chicago data, Dovetail HiC library sequences were aligned and scaffolded following the same method. After scaffolding, shotgun sequences were used to close gaps between contigs.

### Gene model and repetitive elements prediction

The gene model was predicted as described in the previously published Hong Kong oyster (*Magallana hongkongensis*) genome paper [84]. Briefly, the gene models were trained and predicted by funannotate (v1.7.4,https://github.com/nextgenusfs/funannotate) [85] with parameters “–repeats2evm –protein_evidence uniprot_sprot.fasta –genemark_mode ET –busco_seed_species fly –optimize_augustus –busco_db arthropoda –organism other –max_intronlen 350,000”, the gene models from several prediction sources including GeneMark, high-quality Augustus predictions (HiQ), pasa, Augustus, GlimmerHM, and snap were passed to Evidence Modeler to generate the gene model annotation files, PASA was then used to update the EVM consensus predictions, UTR annotations and models for alternatively spliced isoforms were added. The protein-coding gene models were then interrogated using blastp with the nr and swissprot databases in diamond (v0.9.24) with the parameters “–more-sensitive –evalue 1e-3”, and then mapped by HISAT2 (version 2.1.0) with transcriptome reads. Gene matrix count tables were then generated by stringtie (version 2.1.1) and used for further gene expression analyses. Gene models with no homology to any known protein in the GenBank nr database and no mRNA support were removed from the final version. Repetitive elements were predicted as previously described [45].

### Identification of orthologous gene families and macrosynteny analysis

Orthologues and orthogroups in the proteomes of 22 other lepidopteran species (*Amyelois transitella*, *Bicyclus anynana*, *Bombyx mori*, *Calycopis cecrops*, *Chilo suppressalis*, *Danaus plexippus*, *Heliconius erato demophoon*, *Leptidea sinapis*, *Lerema accius*, *Manduca sexta*, *Maniola hyperantus*, *Operophtera brumata*, *Papilio glaucus*, *Papilio machaon*, *Papilio polytes*, *Papilio xuthus*, *Phoebis sennae*, *Pieris macdunnoughi*, *Pieris rapae*, *Plodia interpunctella*, *Plutella xylostella* and *Zerene cesonia*) and *Drosophila melanogaster* were retrieved from NCBI and Lepbase [86], and inferred using OrthoFinder v. 2.5.2 [87] with default values and ‘-M msa’ activated. To symbolize the gene families, the longest protein of each gene was taken as the representative in OrthoFinder analysis. Gene gain and loss rates were computed from gene families using CAFE5 [88]. Single-copy orthologues were anchored by mutual best Diamond blastp hits (–evalue 0.001) between each species pairs. Oxford synteny plots were generated following previously described method [89] using R package ‘ggplot2’ [90].

### Functional terms enrichment analysis

The functional term annotations were performed using eggNOG [91]. Orthogroups were assigned with Gene Ontology (GO), EuKaryotic Orthologous Groups (KOG), Kyoto Encyclopedia of Genes and Genomes (KEGG), and KEGG Orthology (KO) terms by inheriting the terms from genes found within the groups. Functional enrichment was tested using function ‘compareCluster()’ in R package ‘clusterProfiler’ v.4.2.2 [92] under the environment of R 4.1.0 [93]. Significantly enriched terms were determined with pvalueCutoff = 0.05, pAdjustMethod = ‘BH’, and qvalueCutoff = 0.2. The genome of internal nodes (ancestral populations) was inferred according to the gene count results of CAFE5. Data was visualized using R packages ‘ggplot2’ [90], ‘ggtree’ [94] and ‘pathview’ [95].

### Specific gene family annotation and gene tree building

Gene family sequences were first retrieved from the butterfly genome with the use of tBLASTn, in reference to the sequences from species including *D. melanogaster* and *Bombyx mori* via data from the National Center for Biotechnology Information (NCBI) [96]. The retrieved genes were then compared to sequences found in the NCBI nr database with the use of BLASTx to examine their identities. For neuropeptides, amino acid sequences identified in *B. mori* [97], *Spodoptera frugiperda* [98], *Atrijuglans hetaohei* [99], *Cydia pomonella* [100], *Chilo suppressalis* [101], and *Phauda fammans* [102] were used as queries in TBLASTN searches of the *E. hebeca* genome and transcriptomes. Signal sequences were predicted using the SignalP 3.0 Server [103] (http://www.cbs.dtu.dk/services/SignalP). Potential peptide processing sites within the prepropeptides were identified using guidelines outlined in previous study [104]. For the phylogenetic analyses of gene families, DNA sequences were first translated into amino acid sequences and were aligned to the sequences of the members of the respective gene family. Gapped sites were removed from the alignments with the use of MEGA7.0. Phylogenetic trees were constructed with MEGA7.0. Maximum likelihood gene tree was constructed using IQ-TREE [105] with ‘-T AUTO -B 1000 -bnni –alrt 1000’. Gene trees were visualized using R package ‘ggtree’ [94].

### Sex-specific temperature challenge, mRNA and small RNA transcriptome analyses

Adult *E. hecabe* of both sexes (3 butterflies per condition) were exposed to different temperatures (18 °C, 25 °C, and 30 °C) respectively for 24 h continuously with a humidity at around 50% and a cycle of 14 h of light and 10 h of darkness. Total RNA of the treated individuals was extracted separately with the use of the *mir*Vana miRNA Isolation Kit (Invitrogen) following the manufacturer’s instructions. Extracted RNA was subjected to quality control with gel electrophoresis and the Nanodrop spectrophotometer (Thermo Scientific). Samples that were qualified were sent out for transcriptome and sRNA library preparation and sequencing.

The transcriptome reads were separately aligned back to the genome guide trinity assembly generated in the previous gene model prediction step for downstream analyses of differential expression with the script of align_and_estimate_abundance.pl (Trinity version 2.9.1). Gene matrix tables were used for different gene expression analyses by edgeR [106] with default parameters, at least 2 of replicates must have cpm values > 1 cpm value (–min_reps_min_cpm 2,1). Predicted miRNAs were manually examined to identify their identities. In order to identify the conserved miRNAs, the predicted miRNA hairpins from the butterflies were compared to miRNA precursor sequences found on miRbase with the use of BLASTn [107, 108]. Hairpin sequences that had no significant similarity to any miRNA sequences on miRbase were subjected to further manual checking on whether they fulfilled the criteria of miRNAs based on the information on MirGeneDB 2.0 [109]. Sequences that fulfilled the criteria were considered as novel miRNAs.

### Supplementary Information

**Additional file 1: Figure S1.** GenomeScope v2.0 k-mer profile plot based on 21-mers in Illumina reads. The observed k-mer frequency distribution is depicted in blue, whereas the GenomeScope fitmodel is shown as a black line. The unique and putative error k-mer distributions are plotted in yellow and red, respectively. **Figure S2.** Oxford dot plots of orthologous genes between the different lepidopteran species. Orthologous genes are coloured according to their positions in the reference species at the horizontal axis. **Figure S3.** Transposable elements. **Figure S4.** Prcomp principal components and heatmaps of all samples. **Figure S5.** Differentially expressed gene clusters of different combinations of *E. hecabe* sexes and tissues under different heat-stress experiment. Genes differentially expressed between different temperatures (18°C, 25°C and 30°C) in different tissues (H: head; B: body) of both sexes (F: female; M: male) were identified from strand-specific RNA-Seq using EdgeR with three biological replicates (each replicate annotated with number 1, 2 and 3) per sample. **Figure S6.** Differentially expressed protein-coding genes under various temperature settings. Venn diagrams showing the numbers of common and sex-specific differential expressed protein-coding genes when comparing the expression at 18°C with 25°C, 25°C with 30°C and 18°C with 30°C. **Figure S7.** GO annotation of differentially expressed genes in female *Eurema* *hecabe* at 30°C when comparing to 18°C. **Figure S8.** GO annotation of differentially expressed genes in male *Eurema* *hecabe* at 30°C when comparing to 18°C. **Figure S9.** GO annotation of differentially expressed genes in female *Eurema* *hecabe* at 30°C when comparing to 25°C. **Figure S10.** Differentially expressed microRNAs at different temperatures. Venn diagrams showing the numbers of common and sex-specific differential expressed miRNAs when comparing the expression at 18°C with 25°C and 18°C with 30°C. **Figure S11.** Neuropeptides in *E.* *hecabe*. (A) The neuropeptide genes identified in the *E.* *hecabe* genome and their expression in different life stages. (B) Expression of neuropeptide genes under different temperatures. F: female; M, male; H: head; B: body.

**Additional file 2. **Transposable elements information.

**Additional file 3. **Summary of differentially expressed protein coding genes and microRNAs.

**Additional file 4. **Expression data of mRNA.

**Additional file 5. **Gene ontology and pathway enrichment analyses data.

**Additional file 6. **Expression data of sesquiterpenoid pathway genes.

**Additional file 7. **Expression data of microRNAs.

**Additional file 8. **Expression data of neuropeptides

**Additional file 9. **Comparative sex-biased analysis.

**Additional file 10.** Sequencing data.

## Data Availability

The raw reads generated in this study have been deposited to the NCBI database under the BioProject accession PRJNA664668 [110]. The final chromosome assembly was submitted to NCBI Assembly under accession number JADANM000000000 in NCBI [111]. The genome annotation files were deposited in the Figshare [112].

## Abbreviations

AKH: Adipokinetic hormone
BRh: Blue-sensitive rhodopsin
BUSCO: Benchmarking universal single-copy orthologs
CHH: Crustacean hyperglycemic hormone
COI: Cytochrome oxidase I
FPPS: Farnesyl diphosphate synthase
LINE: Long interspersed nuclear element
LTR: Long terminal repeats
mRNA: Messenger RNA
MVK: Mevalonate kinase
NEURL4: Neuralized-like protein 4
pburs: Partner of bursicon
PTTH: Prothoracicotropic hormone
SINE: Short interspersed nuclear elements
sNPF: Short neuropeptide F
sRNA: Small RNA
TE: Transposable element
Tret-1: Facilitated trehalose transporter Tret1-like
UVRh1: Ultraviolet opsin gene 1
