# Supplementary material for: The genome and sex-dependent responses to temperature in the common yellow butterfly, Eurema hecabe
Source: BMC Biol. 2023 Sep 25;21:200. doi: 10.1186/s12915-023-01703-1 (PMC10521528; doi:10.1186/s12915-023-01703-1)
Supplement: Supplementary file 1 — Additional file 1: Figure S1. GenomeScope v2.0 k-mer profile plot based on 21-mers in Illumina reads. The observed k-mer frequency distribution is depicted in blue, whereas the GenomeScope fitmodel is shown as a black line. The unique and putative error k-mer distributions are plotted in yellow and red, respectively. Figure S2. Oxford dot plots of orthologous genes between the different lepidopteran species. Orthologous genes are coloured according to their positions in the reference species at the horizontal axis. Figure S3. Transposable elements. Figure S4. Prcomp principal components and heatmaps of all samples. Figure S5. Differentially expressed gene clusters of different combinations of E. hecabe sexes and tissues under different heat-stress experiment. Genes differentially expressed between different temperatures (18°C, 25°C and 30°C) in different tissues (H: head; B: body) of both sexes (F: female; M: male) were identified from strand-specific RNA-Seq using EdgeR with three biological replicates (each replicate annotated with number 1, 2 and 3) per sample. Figure S6. Differentially expressed protein-coding genes under various temperature settings. Venn diagrams showing the numbers of common and sex-specific differential expressed protein-coding genes when comparing the expression at 18°C with 25°C, 25°C with 30°C and 18°C with 30°C. Figure S7. GO annotation of differentially expressed genes in female Eurema hecabe at 30°C when comparing to 18°C. Figure S8. GO annotation of differentially expressed genes in male Eurema hecabe at 30°C when comparing to 18°C. Figure S9. GO annotation of differentially expressed genes in female Eurema hecabe at 30°C when comparing to 25°C. Figure S10. Differentially expressed microRNAs at different temperatures. Venn diagrams showing the numbers of common and sex-specific differential expressed miRNAs when comparing the expression at 18°C with 25°C and 18°C with 30°C. Figure S11. Neuropeptides in E. hecabe. (A) The neuropeptide [file 12915_2023_1703_MOESM1_ESM.pdf]

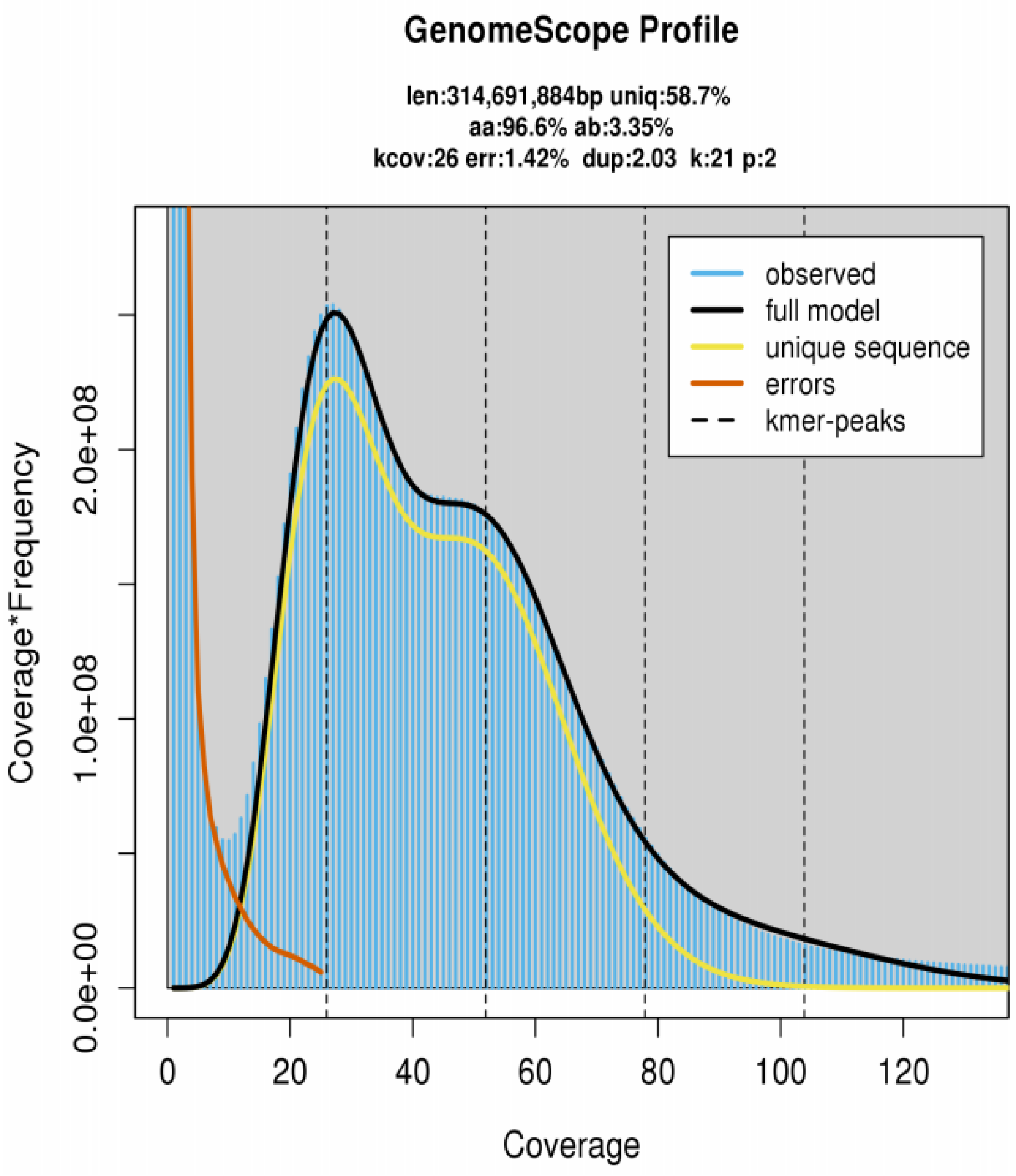
GenomeScope Profile
len:314,691,884bp uniq:58.7%
aa:96.6% ab:3.35%
kcov:26 err:1.42% dup:2.03 k:21 p:2
observed
full model
unique sequence
errors
kmer-peaks
Coverage*Frequency
0.0e+00
1.0e+08
2.0e+08
0
20
40
60
80
100
120
Coverage

Figure S1

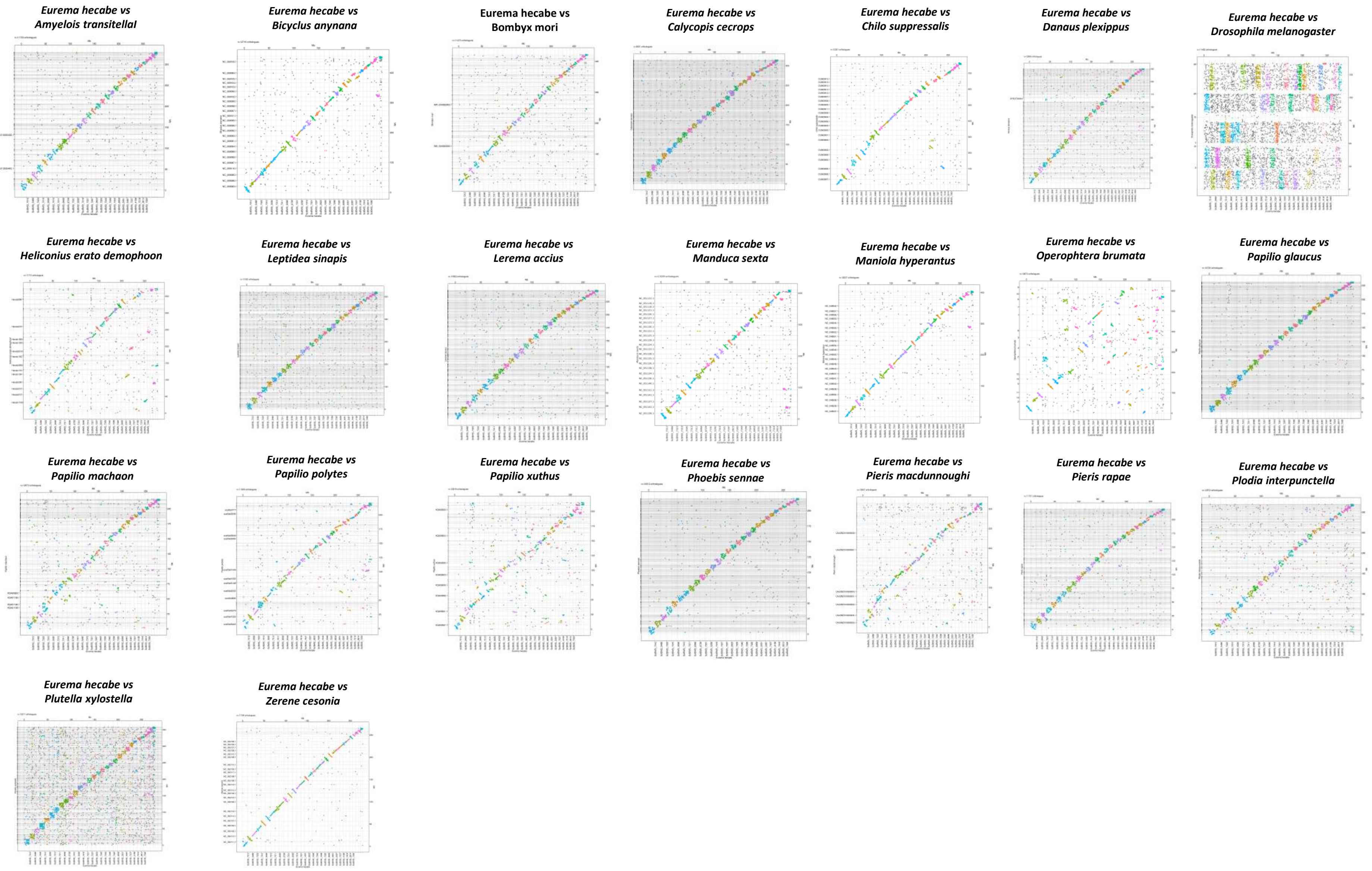

High resolution figures can be found at
https://doi.org/10.6084/m9.figshare.19634646

Figure S2

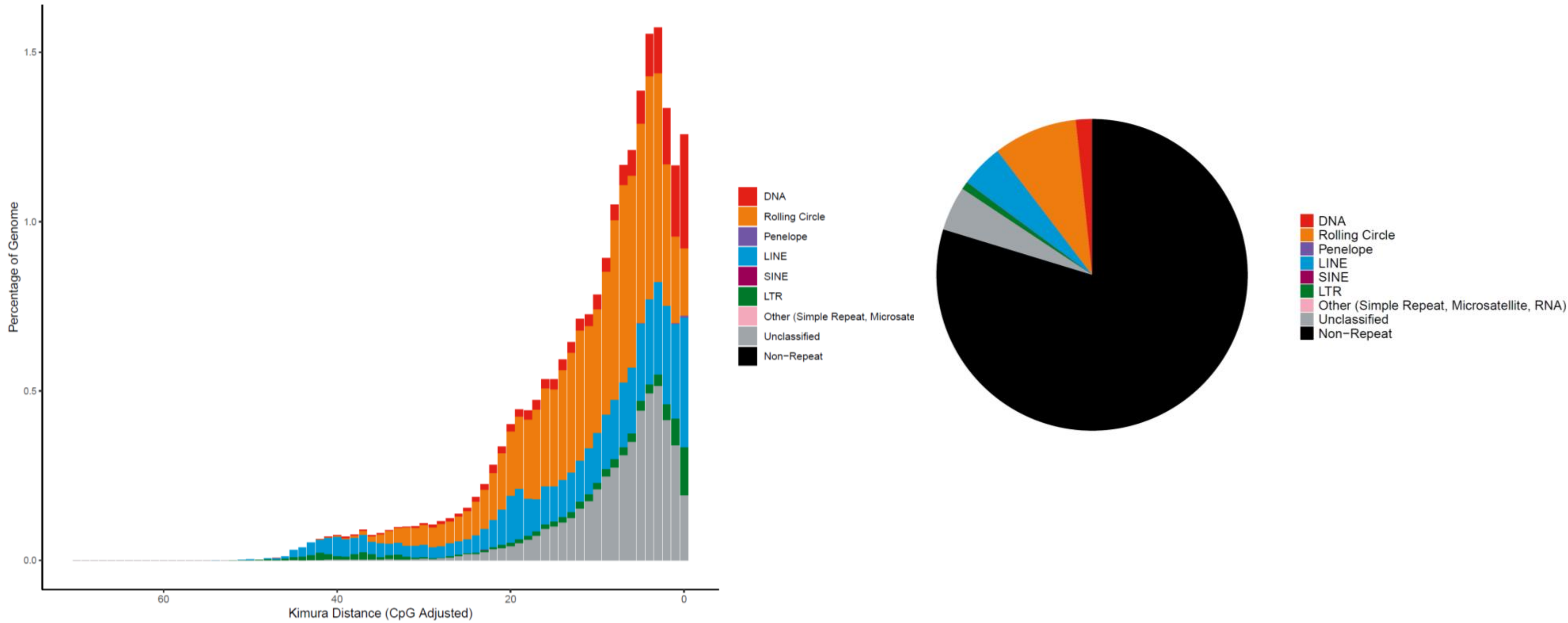

1.5
1.0
0.5
0.0
Percentage of Genome
60
40
20
0
Kimura Distance (CpG Adjusted)
DNA
Rolling Circle
Penelope
LINE
SINE
LTR
Other (Simple Repeat, Microsate
Unclassified
Non−Repeat
DNA
Rolling Circle
Penelope
LINE
SINE
LTR
Other (Simple Repeat, Microsatellite, RNA)
Unclassified
Non−Repeat

Figure S3

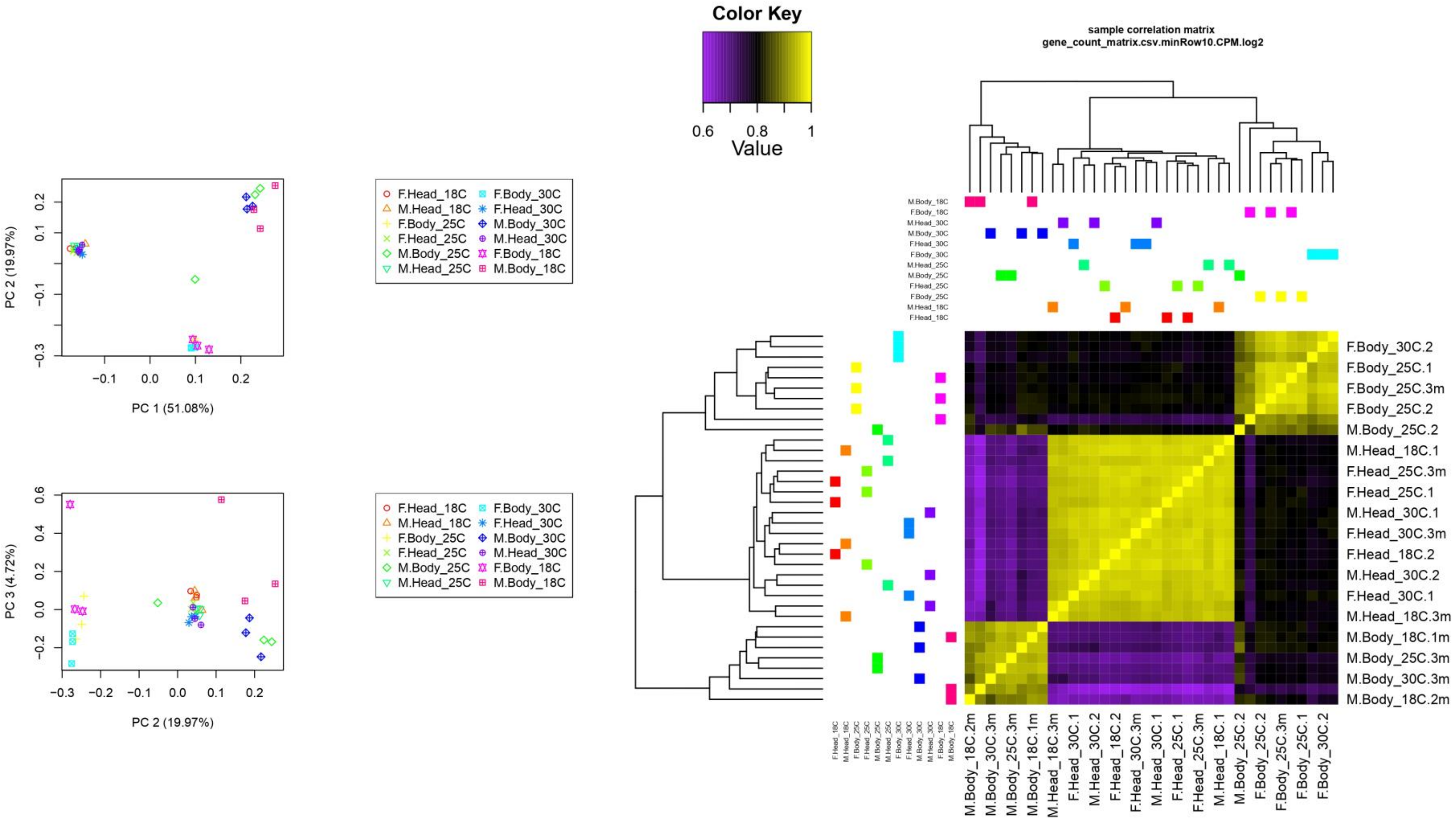

PC 2 (19.97%)
PC 1 (51.08%)
PC 3 (4.72%)
PC 2 (19.97%)
F.Head_18C
M.Head_18C
F.Body_25C
F.Head_25C
M.Body_25C
M.Head_25C
F.Body_30C
F.Head_30C
M.Body_30C
M.Head_30C
F.Body_18C
M.Body_18C
Color Key
0.6 0.8 1
Value
sample correlation matrix
gene_count_matrix.csv.minRow10.CPM.log2
F.Body_30C.2
F.Body_25C.1
F.Body_25C.3m
F.Body_25C.2
M.Body_25C.2
M.Head_18C.1
F.Head_25C.3m
F.Head_25C.1
M.Head_30C.1
F.Head_30C.3m
F.Head_18C.2
M.Head_30C.2
F.Head_30C.1
M.Head_18C.3m
M.Body_18C.1m
M.Body_25C.3m
M.Body_30C.3m
M.Body_18C.2m

Figure S4

male_head_edgeR_gene.min_reps2.min_cpm1

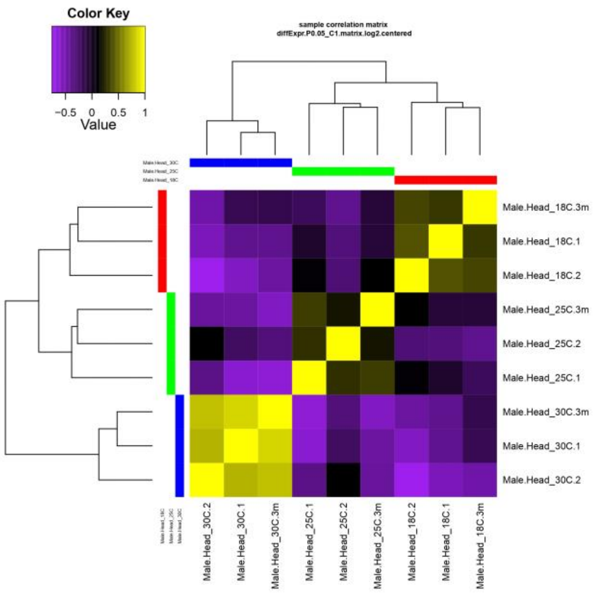

male_body_edgeR_gene.min_reps2.min_cpm1

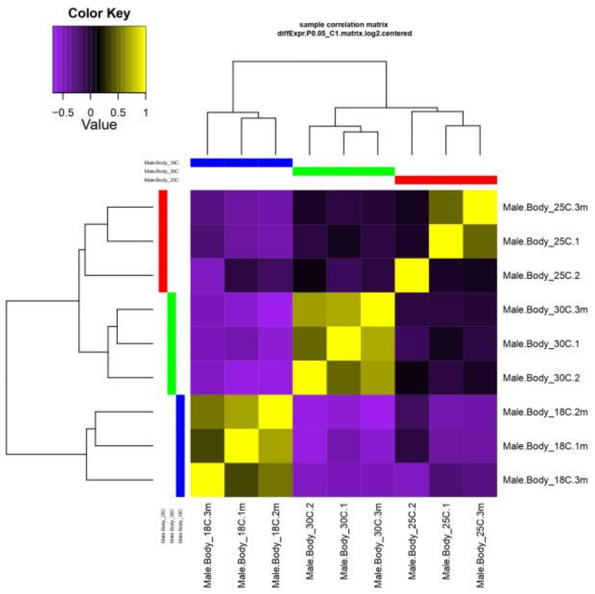

female_head_edgeR_gene.min_reps2.min_cpm1

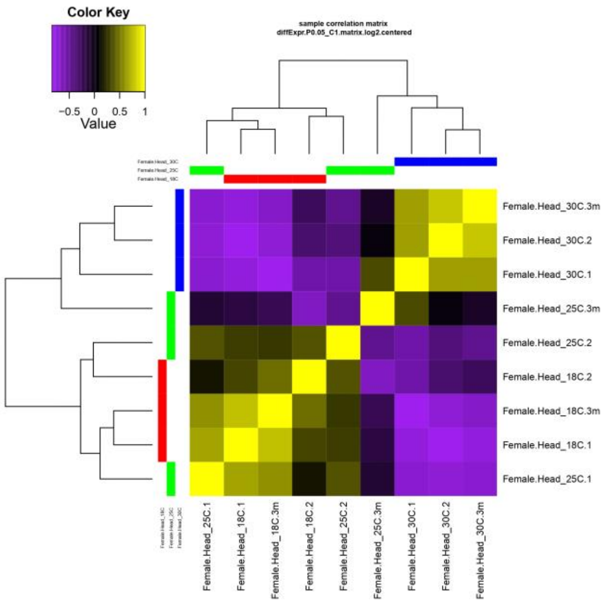

female_body_edgeR_gene.min_reps2.min_cpm1

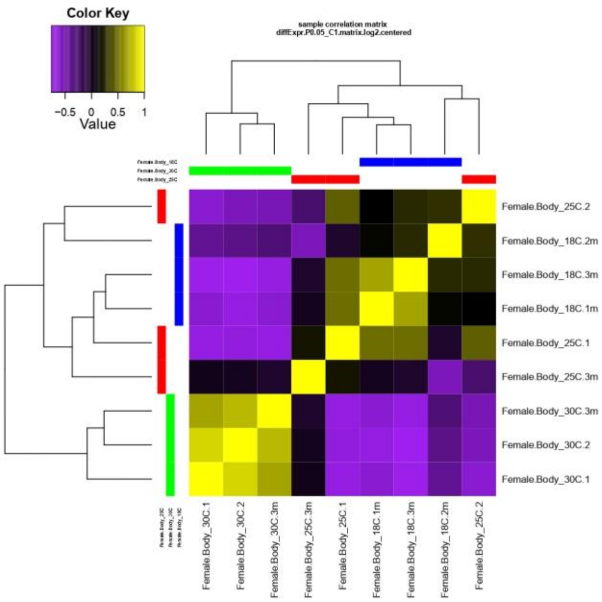

Figure S5

Differential expressed genes at different temperature settings

30°C vs 25°C

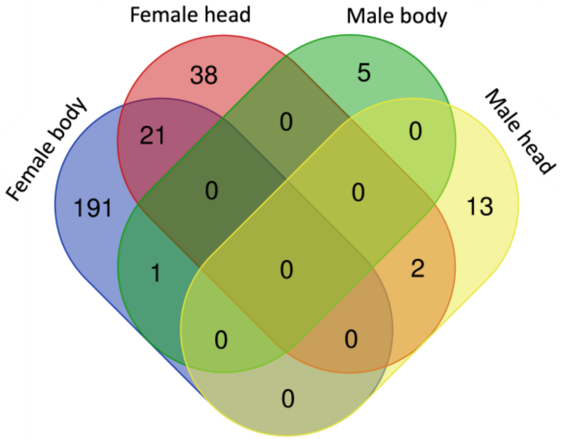

18°C vs 25°C

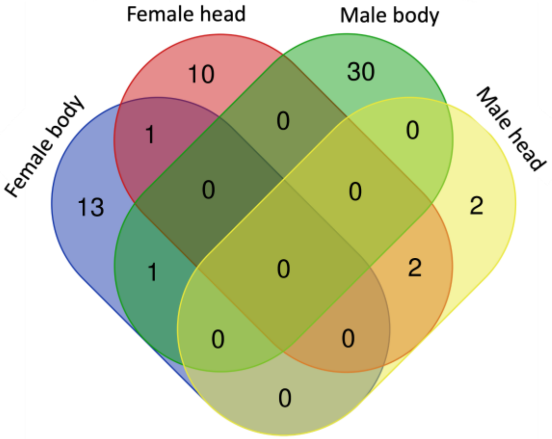

30°C vs 18°C

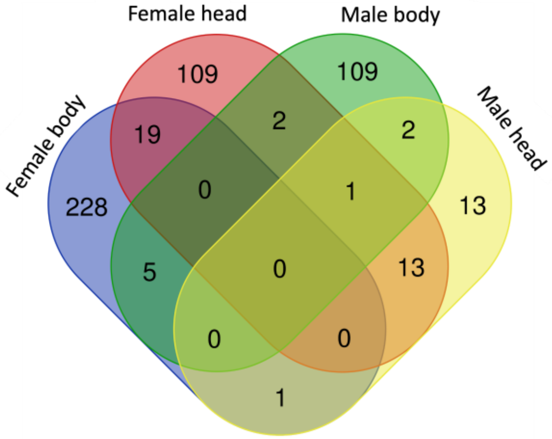

Figure S6

18C vs 30C (Female)

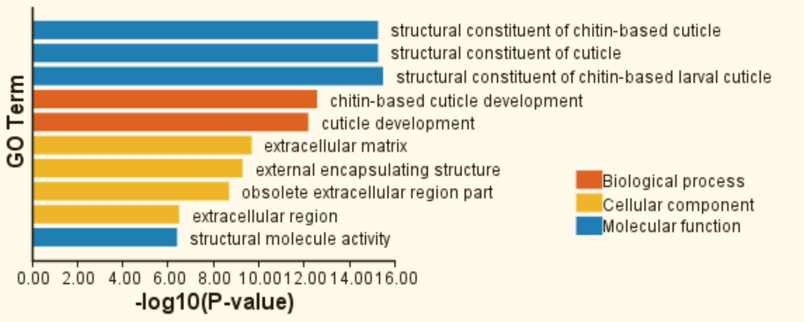

Down-regulated at 30°C in female's body

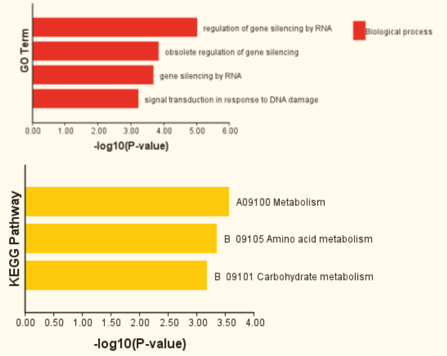

Up-regulated at 30°C in female's head

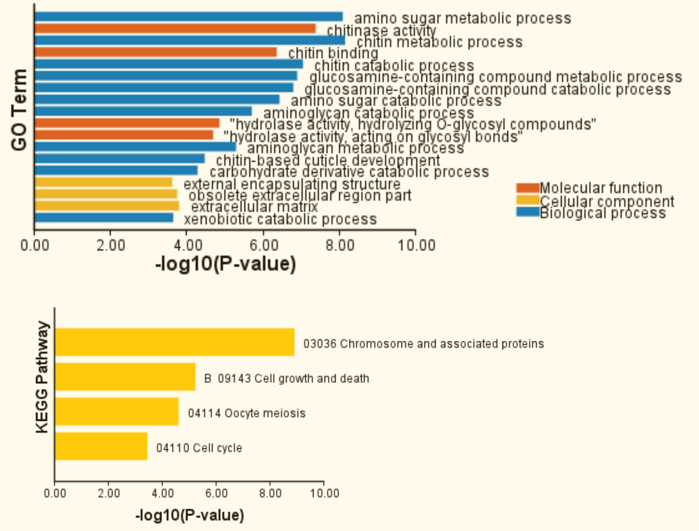

Down-regulated at 30°C in female's head

Figure S7

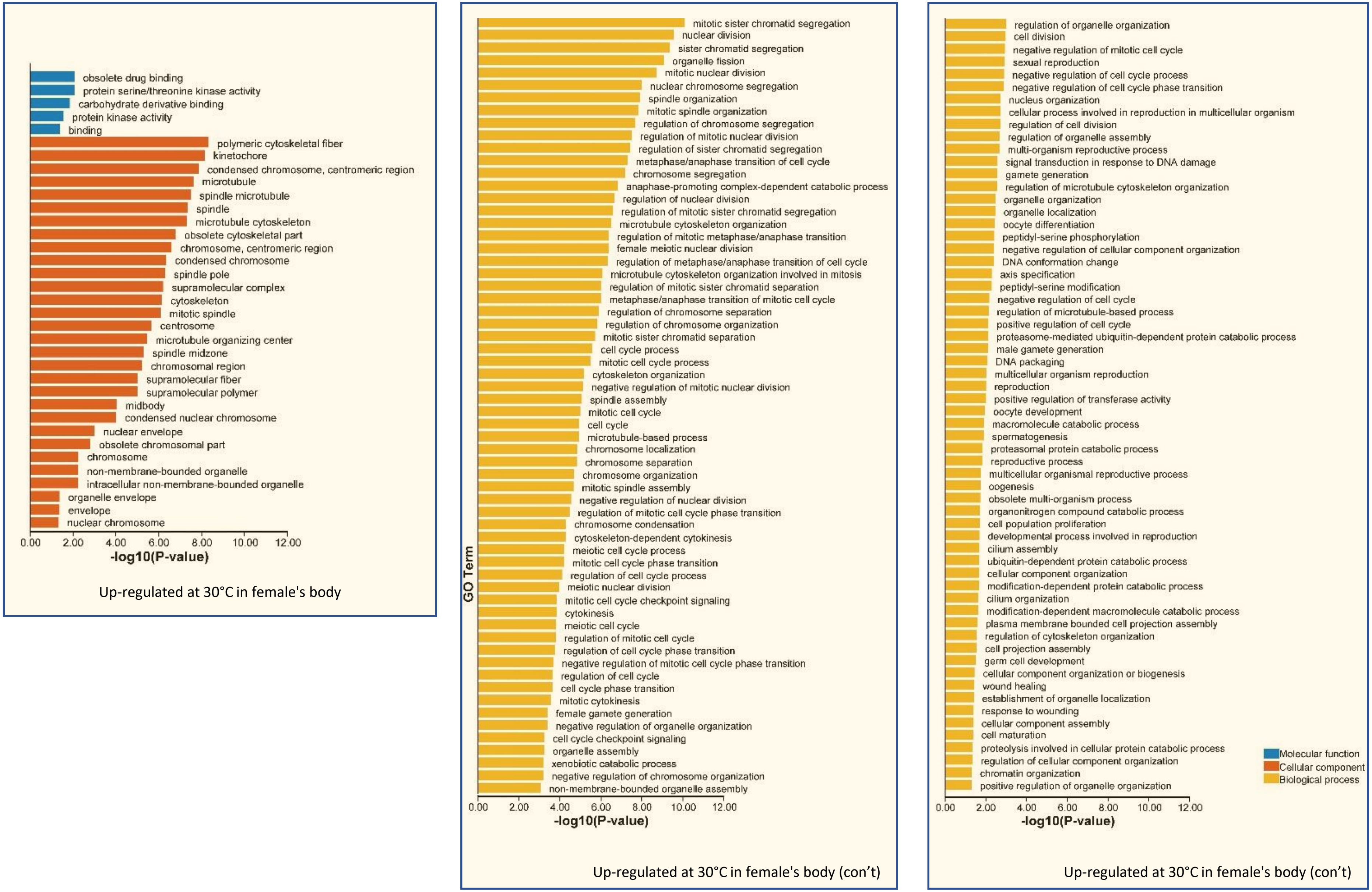

Up-regulated at 30°C in female's body

Up-regulated at 30°C in female's body (con't)

Up-regulated at 30°C in female's body (con't)

Figure S7

18C vs 30C (Male)

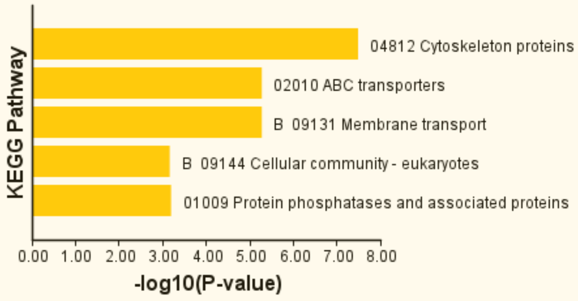

Up-regulated at 30°C in male's head

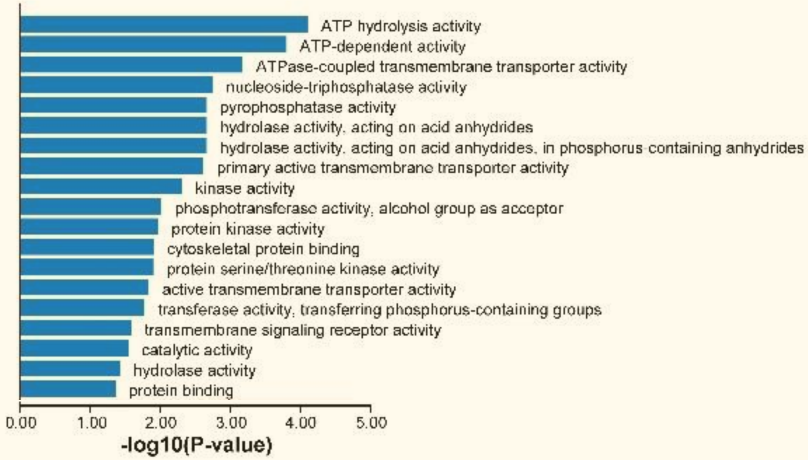

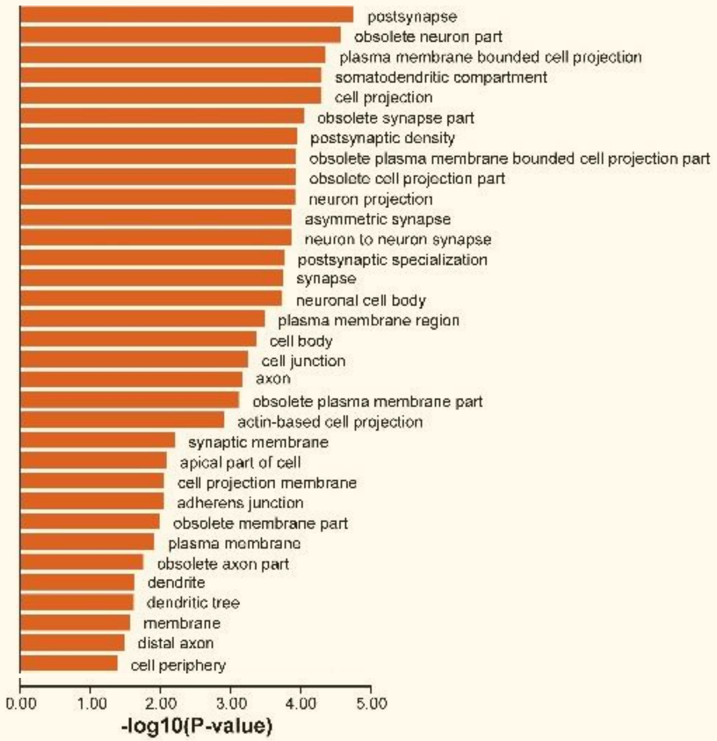

Up-regulated at 30°C in male's body

Figure S8

18C vs 30C (Male)

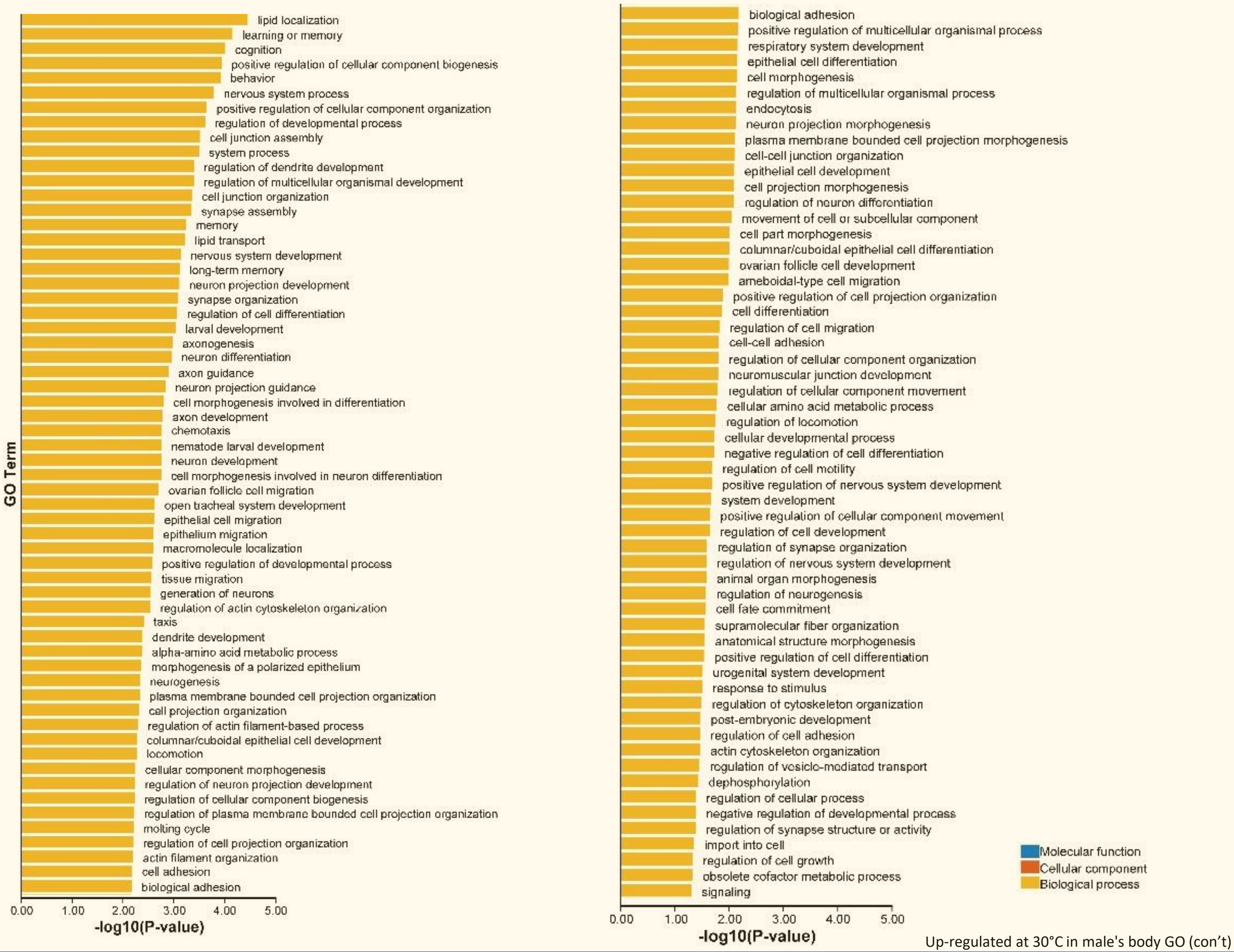

Up-regulated at 30°C in male's body GO (con't)

Figure S8

25C vs 30C (Female)

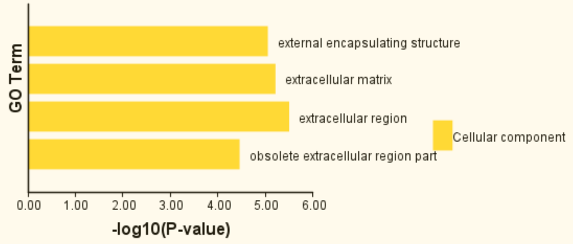

Down-regulated at 30°C in female's head

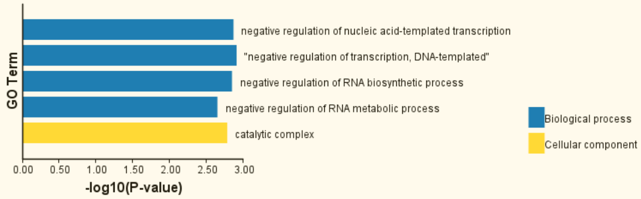

Up-regulated at 30°C in female's head

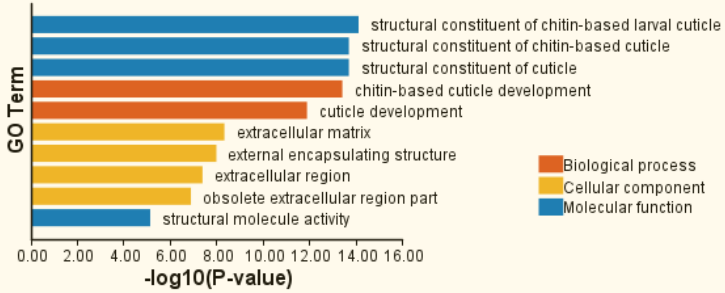

Down-regulated at 30°C in female's body

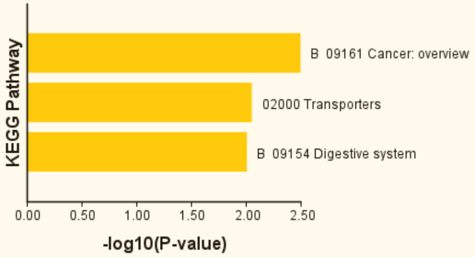

Up-regulated at 30C in female's head

Figure S9

Differential expressed microRNAs at different temperature settings

18°C vs 25°C

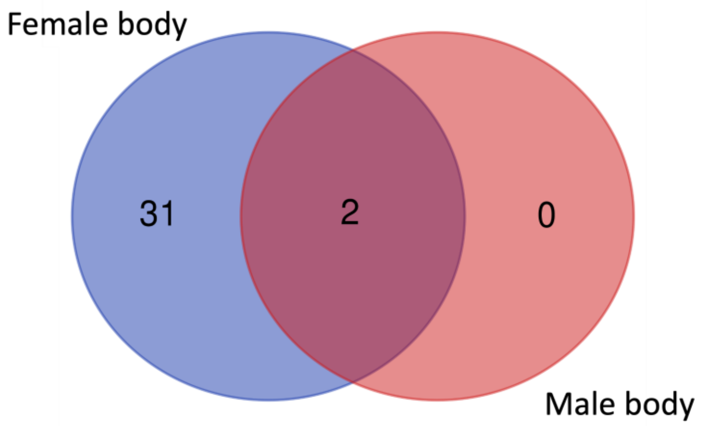

18°C vs 30°C

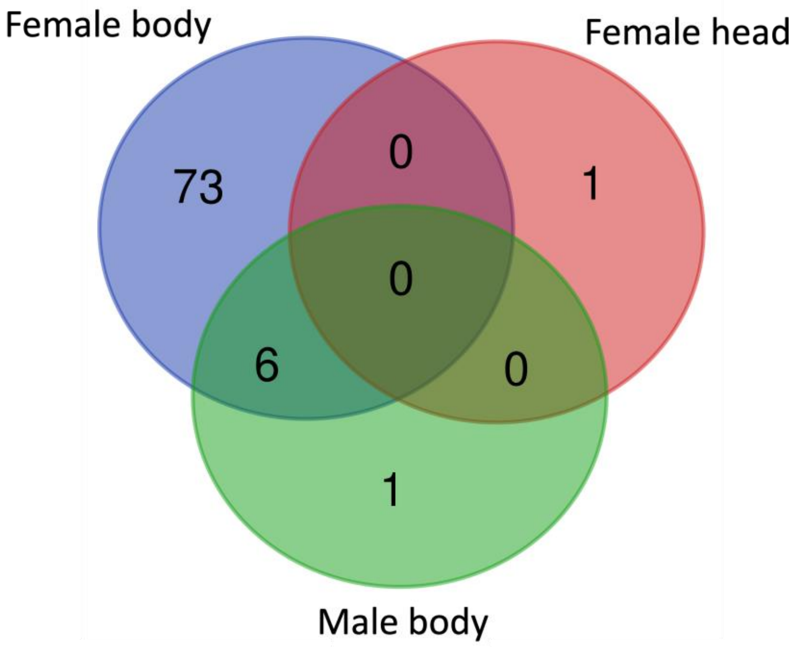

Figure S10

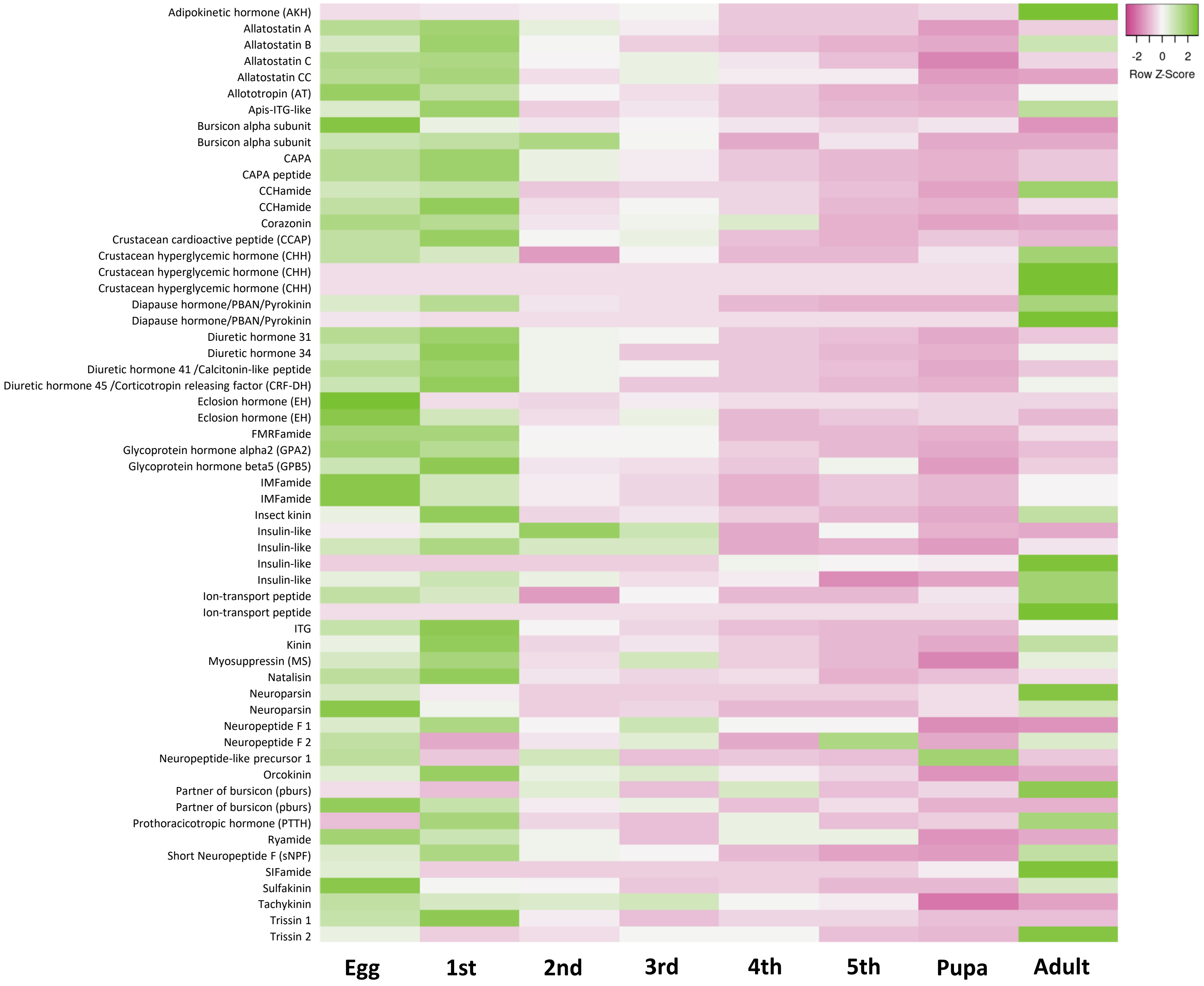
Adipokinetic hormone (AKH)
Allatostatin A
Allatostatin B
Allatostatin C
Allatostatin CC
Allototropin (AT)
Apis-ITG-like
Bursicon alpha subunit
Bursicon alpha subunit
CAPA
CAPA peptide
CCHamide
CCHamide
Corazonin
Crustacean cardioactive peptide (CCAP)
Crustacean hyperglycemic hormone (CHH)
Crustacean hyperglycemic hormone (CHH)
Crustacean hyperglycemic hormone (CHH)
Diapause hormone/PBAN/Pyrokinin
Diapause hormone/PBAN/Pyrokinin
Diuretic hormone 31
Diuretic hormone 34
Diuretic hormone 41 /Calcitonin-like peptide
Diuretic hormone 45 /Corticotropin releasing factor (CRF-DH)
Eclosion hormone (EH)
Eclosion hormone (EH)
FMRFamide
Glycoprotein hormone alpha2 (GPA2)
Glycoprotein hormone beta5 (GPB5)
IMFamide
IMFamide
Insect kinin
Insulin-like
Insulin-like
Insulin-like
Insulin-like
Ion-transport peptide
Ion-transport peptide
ITG
Kinin
Myosuppressin (MS)
Natalisin
Neuroparsin
Neuroparsin
Neuropeptide F 1
Neuropeptide F 2
Neuropeptide-like precursor 1
Orcokinin
Partner of bursicon (pburs)
Partner of bursicon (pburs)
Prothoracicotropic hormone (PTTH)
Ryamide
Short Neuropeptide F (sNPF)
SIFamide
Sulfakinin
Tachykinin
Trissin 1
Trissin 2
Egg
1st
2nd
3rd
4th
5th
Pupa
Adult
-2
0
2
Row Z-Score

Figure S11

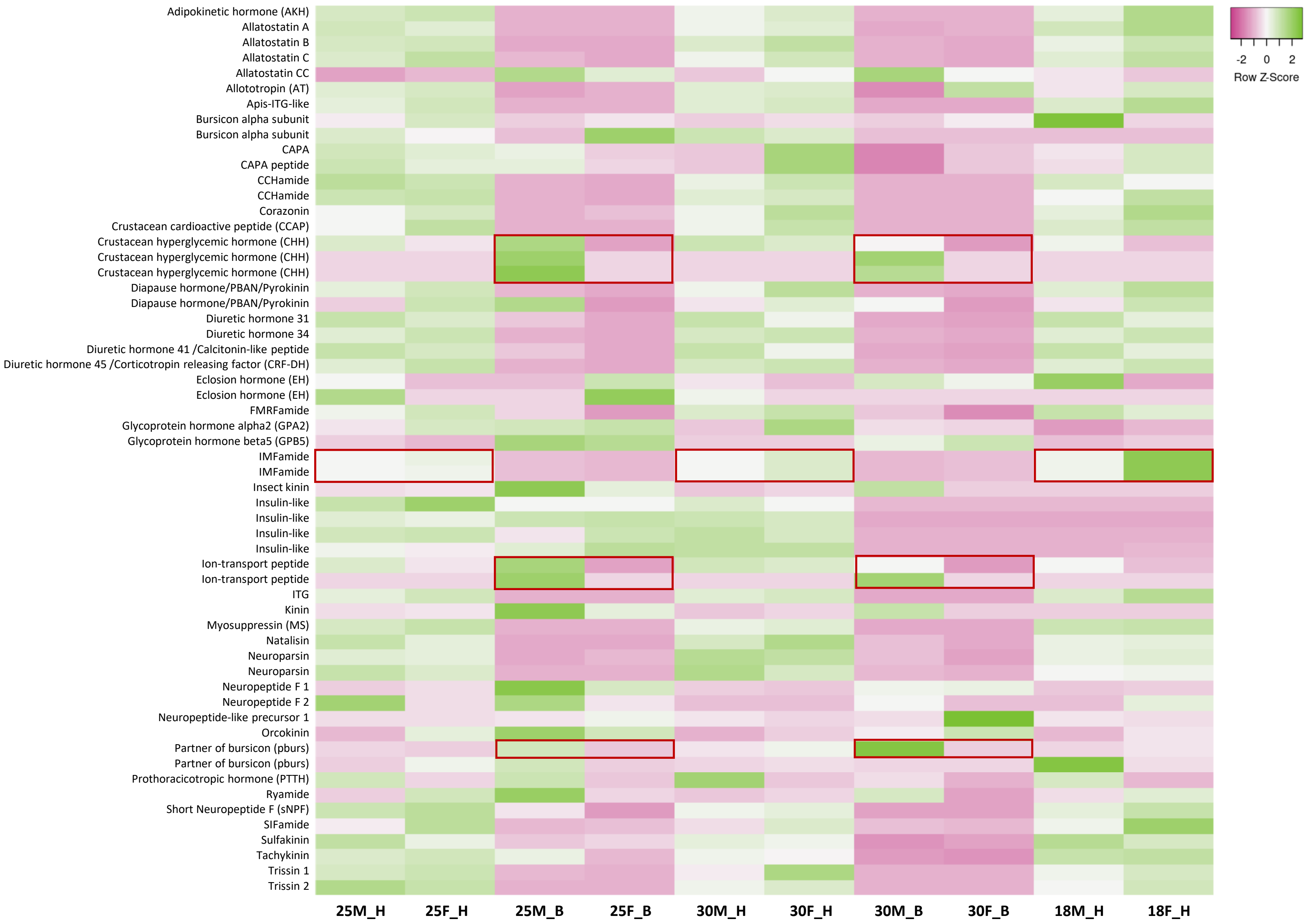
Adipokinetic hormone (AKH)
Allatostatin A
Allatostatin B
Allatostatin C
Allatostatin CC
Allototropin (AT)
Apis-ITG-like
Bursicon alpha subunit
Bursicon alpha subunit
CAPA
CAPA peptide
CCHamide
CCHamide
Corazonin
Crustacean cardioactive peptide (CCAP)
Crustacean hyperglycemic hormone (CHH)
Crustacean hyperglycemic hormone (CHH)
Crustacean hyperglycemic hormone (CHH)
Diapause hormone/PBAN/Pyrokinin
Diapause hormone/PBAN/Pyrokinin
Diuretic hormone 31
Diuretic hormone 34
Diuretic hormone 41 /Calcitonin-like peptide
Diuretic hormone 45 /Corticotropin releasing factor (CRF-DH)
Eclosion hormone (EH)
Eclosion hormone (EH)
FMRFamide
Glycoprotein hormone alpha2 (GPA2)
Glycoprotein hormone beta5 (GPB5)
IMFamide
IMFamide
Insect kinin
Insulin-like
Insulin-like
Insulin-like
Insulin-like
Ion-transport peptide
Ion-transport peptide
ITG
Kinin
Myosuppressin (MS)
Natalisin
Neuroparsin
Neuroparsin
Neuropeptide F 1
Neuropeptide F 2
Neuropeptide-like precursor 1
Orcokinin
Partner of bursicon (pburs)
Partner of bursicon (pburs)
Prothoracicotropic hormone (PTTH)
Ryamide
Short Neuropeptide F (sNPF)
SIFamide
Sulfakinin
Tachykinin
Trissin 1
Trissin 2
25M_H
25F_H
25M_B
25F_B
30M_H
30F_H
30M_B
30F_B
18M_H
18F_H
-2
0
2
Row Z-Score

Figure S11
